# The Functional Mammalian CRES (Cystatin-Related Epididymal Spermatogenic) Amyloid is Antiparallel β-Sheet Rich and Forms a Metastable Oligomer During Assembly

**DOI:** 10.1038/s41598-019-45545-w

**Published:** 2019-06-25

**Authors:** Hoa Quynh Do, Aveline Hewetson, Caitlyn Myers, Nazmul H. Khan, Mary Catherine Hastert, Faraz M. Harsini, Michael P. Latham, Benjamin J. Wylie, R. Bryan Sutton, Gail A. Cornwall

**Affiliations:** 1 Department of Cell Biology and Biochemistry, Texas Tech University Health Sciences Center, Lubbock, TX USA; 2 Department of Cell Physiology and Molecular Biophysics, Texas Tech University Health Sciences Center, Lubbock, TX USA; 3 College of Arts and Sciences Microscopy, Texas Tech University, Lubbock, TX USA; 4 Department of Chemistry and Biochemistry, Texas Tech University, Lubbock, TX USA

**Keywords:** Solid-state NMR, Protein aggregation, Reproductive biology, Protein aggregation

## Abstract

An amyloid matrix composed of several family 2 cystatins, including the reproductive cystatin CRES, is an integral structure in the mouse epididymal lumen and has proposed functions in sperm maturation and protection. Understanding how CRES amyloid assembles *in vitro* may provide clues on how the epididymal amyloid matrix forms *in vivo*. We therefore purified full-length CRES under nondenaturing conditions and followed its aggregation from monomer to amyloid under conditions that may approximate those in the epididymal lumen. CRES transitioned into a metastable oligomer that was resistant to aggregation and only over extended time formed higher-ordered amyloids. High protein concentrations facilitated oligomer assembly and also were required to maintain the metastable state since following dilution the oligomer was no longer detected. Similar to other amyloid precursors, the formation of CRES amyloids correlated with a loss of α-helix and a gain of β-sheet content. However, CRES is unique in that its amyloids are rich in antiparallel β-sheets instead of the more common parallel β-sheets. Taken together, our studies suggest that early metastable oligomers may serve as building blocks for functional amyloid assembly and further reveal that antiparallel β-sheet-rich amyloids can be functional forms.

## Introduction

The epididymis is a long convoluted tubule in which spermatozoa acquire their functions of motility and fertility as they migrate from the proximal (caput) to distal (cauda) end. The maturation of spermatozoa requires the active involvement of the surrounding epididymal epithelium that secretes proteins into the lumen. These proteins interact with and modify the migrating spermatozoa ultimately resulting in their functional maturation. In addition to maturation, the epididymis must also protect spermatozoa from pathogens that can ascend the male tract and cause transient or permanent infertility. However, the mechanism(s) by which the epididymis carries out these functions is not known.

We previously established that a nonpathological amyloid matrix with putative roles in sperm maturation and protection is a normal component of the mouse epididymal lumen^1^. The amyloid matrix contains multiple members of the family 2 cystatins of cysteine protease inhibitors, all of which are secreted into the lumen by the epididymal epithelium and which colocalize with one another in the amyloid matrix^2^. These include cystatin C and four members of the CRES (cystatin-related epididymal spermatogenic) subgroup (CRES, CRES2, CRES3 cystatin E2), a reproductive subgroup within the family 2 cystatins^2,3^. We further showed that the amyloid forms of CRES subgroup members are present in the epididymal amyloid matrix and that all members readily formed amyloid *in vitro*, suggesting these structures carry out functional roles in the epididymis^2^. Cystatin C is also an established amyloidogenic protein^4^. Human cystatin C has been implicated in Alzheimer’s disease as suggested by the genetic linkage of a cystatin C polymorphism with late onset disease^5,6^ and that cystatin C colocalizes with amyloid-β in plaques associated with Alzheimer’s disease^7^. However, *in vitro* cystatin C inhibited amyloid-β fibril formation^8–10^ and *in vivo* cystatin C inhibited the deposition of amyloid-β in several amyloid precursor protein mouse models^11,12^ implying it plays protective rather than pathological roles. Together, these studies suggest the cystatin amyloids may carry out coordinated biological functions in the epididymal lumen through their organization into a common amyloid matrix. Indeed, CRES, CRES2, and cystatin C exhibit antimicrobial activity *in vitro* suggesting they may have similar functions *in vivo*^13–15^. However, whether these activities are associated with their amyloid forms has not been determined. Using different mouse models we further demonstrated that alterations in the levels of amyloid matrix cystatins, caused either by the loss of CRES in the CRES knockout (KO) mouse or by the overexpression of a highly amyloidogenic mutant (L68Q) cystatin C led to a disrupted amyloid matrix structure and epididymal pathology^16,17^. These conditions included a lysosomal storage-like disease and infertility^16,17^. Thus although emphasizing their roles in normal epididymal functions, these studies also showed that perturbed amyloid matrices can underlie disease states.

Our studies of nonpathological amyloid in the epididymis contributed to a growing body of evidence showing that many amyloids perform biological roles rather than being associated with disease and are known as functional amyloids. These now comprise amyloids that serve as biological scaffolds, signaling complexes, and storage depots and include functions in germline specification, long-term memory, sperm clearance, and fertilization^18–25^. However, because pathological and functional amyloids follow similar aggregation pathways, it remains unclear how functional amyloids assemble into their mature structures *in vivo* and avoid the cytotoxicity that is often associated with the intermediate oligomeric amyloid forms^26^. Several studies, including our previous work, showed that some functional amyloids can assemble within minutes into higher ordered amyloids including fibrils suggesting that the rapid kinetics of their assembly may minimize or avoid potentially cytotoxic oligomers^1,18^. In these reports, however, denatured protein was examined as it was diluted out of high concentrations of guanidine or urea; conditions that likely do not represent the environment in which the amyloid precursor acquires its fold *in vivo*. Further, conclusions of amyloid assembly and toxicity have often been drawn from analyses of individual amyloidogenic domains rather than full-length protein which also does not reflect the *in vivo* situation. Because of these limitations, a clear understanding of the mechanism of functional amyloid assembly is still needed.

We recently established a protocol for the expression and purification of full-length mouse CRES from the soluble fraction of bacteria yielding preparations of a nondenatured protein. This provided us with the means to study the different assembly states of CRES as it transitioned to amyloid under conditions that may more closely approximate those which occur *in vivo*. Herein we show that after purification CRES aggregated from a monomer with mixed secondary structure into a stable antiparallel β-sheet rich oligomer and that protein concentration accelerated this transition. The CRES oligomer was resistant to further assembly and only over extended time transitioned into higher ordered antiparallel β-sheet rich amyloids. However, the addition of CRES amyloid seeds, generated *in vitro* or in the form of endogenous epididymal amyloid matrix facilitated this assembly. Unlike several previously described functional and pathological amyloids, CRES amyloids were not cytotoxic to mammalian cells.

## Results

### Early oligomeric states of CRES

**Figure 1:**
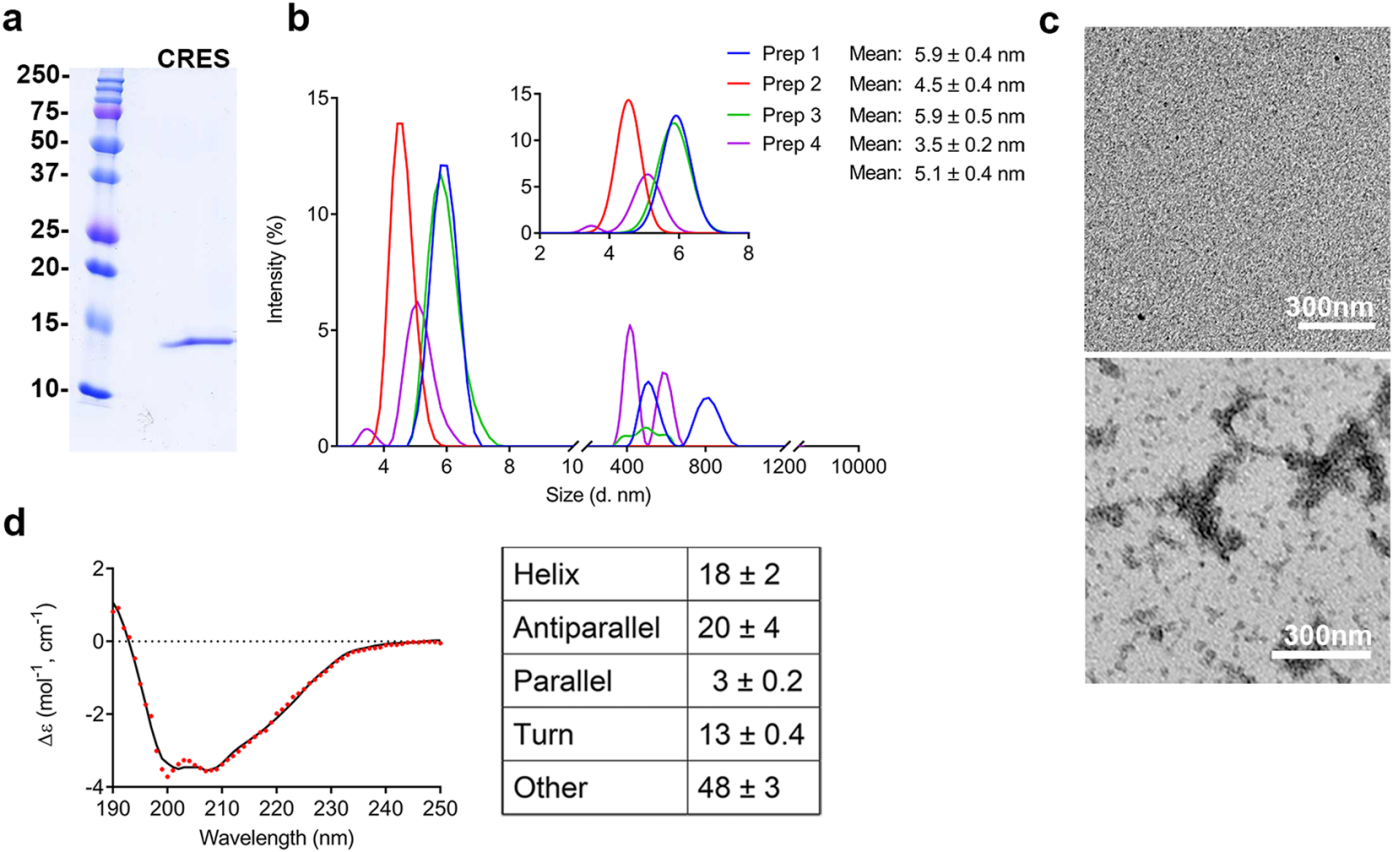
Early oligomeric states of CRES. (**a)** Coomassie Blue stained SDS-PAGE gel of purified CRES showed a single band at approximately 14 kDa. (**b**) DLS analysis of four different CRES preparations (Prep 1–4) (~11–16 μM) in high salt gel filtration buffer. Intensity measurements showed populations of CRES particles at 4–8 nm and 400–1000 nm. Inset, an average hydrodynamic radius for the particles between 4–8 nm was calculated from the fitted data and diameter ± SD is reported. Due to large variations in particle size we were unable to fit the CRES particles between 400–1000 nm. (**c**) Negative stain TEM showed CRES was present as granular structures (top panel) with some oligomeric/protofibril amyloid forms (bottom panel). Data are representative of n = 4 CRES preparations. (**d**) CD analysis of ~11–16 μM CRES exchanged into 4 mM potassium phosphate buffer indicated a protein with mixed secondary structure. CD spectral curve shows experimental (dotted line) and fitted data (solid line) from a representative CRES preparation. Table shows the mean ± SEM secondary structure as predicted from the spectral data by the BeStSel server from n = 3 independent CRES preparations.

Mouse CRES containing a single amino acid substitution, cysteine 48 replaced with alanine (C48A) to prevent inappropriate disulfide bond formation, was expressed as a GST-fusion protein in bacteria. Tagless CRES C48A (CRES) was purified from the soluble fraction of bacteria using affinity, ion exchange, and gel filtration chromatography. Examination of the protein by SDS-PAGE revealed a single protein at the expected molecular weight of 14 kDa showing we had isolated a pure, homogeneous population of full-length CRES (Fig. 1a). Although CRES eluted off the gel filtration column as a single peak that was predicted to contain its monomeric form (Supplementary Fig. S1), dynamic light scattering (DLS), used to determine the size of the purified CRES in solution, showed two distinct populations that varied slightly depending on the protein preparation. The data in Fig. 1b show the intensity distributions from four different protein preparations that were analyzed within 2 hours after elution off the gel filtration column. In all preparations there was a predominant population with a particle size between 4–8 nm and a second population of larger particles between 400–1000 nm. Although we were unable to fit the second population of particles because of their large variation in size, an average hydrodynamic radius was calculated from the fitted data for the particles in the 4–8 nm group and diameter ± SD is reported (Fig. 1b inset). In the four CRES preparations examined, two contained particles with an average diameter of 4.5 ± 0.4 nm and 5.1 ± 0.4 nm whereas the other preparations contained a larger particle of 5.9 ± 0.5 nm. In one preparation, an additional particle size of 3.5 ± 0.2 nm was observed. Based on published reports of the related cystatin C, we believe the 4.5–5.1 nm particle is the CRES monomer while the larger 5.9 nm particle may represent an early CRES aggregate^27^. Indeed, negative stain TEM of the same samples examined by DLS showed the majority of CRES was present as granular material with occasional patches of small balls typical of amyloid oligomers and clusters of short fibrils characteristic of amyloid protofibrils suggesting CRES has a tendency to self-assemble (Fig. 1c). Freshly eluted CRES was buffer exchanged out of the high salt gel filtration buffer into potassium phosphate buffer, pH 7.4, compatible for circular dichroism (CD), and spectra were immediately collected. Secondary structure was predicted from the CD spectral data using the BeStSel server that was designed for β-structure-rich proteins and which reliably distinguishes parallel from antiparallel β-sheets^28^. This analysis showed a protein composed of 18 ± 2% α-helix, 20 ± 4% antiparallel β-sheet, 3 ± 0.2% parallel β-sheet, 13 ± 0.4% turn, and 48 ± 2% other (Fig. 1d). Similar secondary structure composition was predicted using the CONTINLL algorithm through the DichroWeb server^29^, although total β-strands were reported (Supplementary Fig. S2). These results show the early forms of CRES oligomers possess mixed secondary structure.

### CRES assembles into a stable oligomeric intermediate

**Figure 2:**
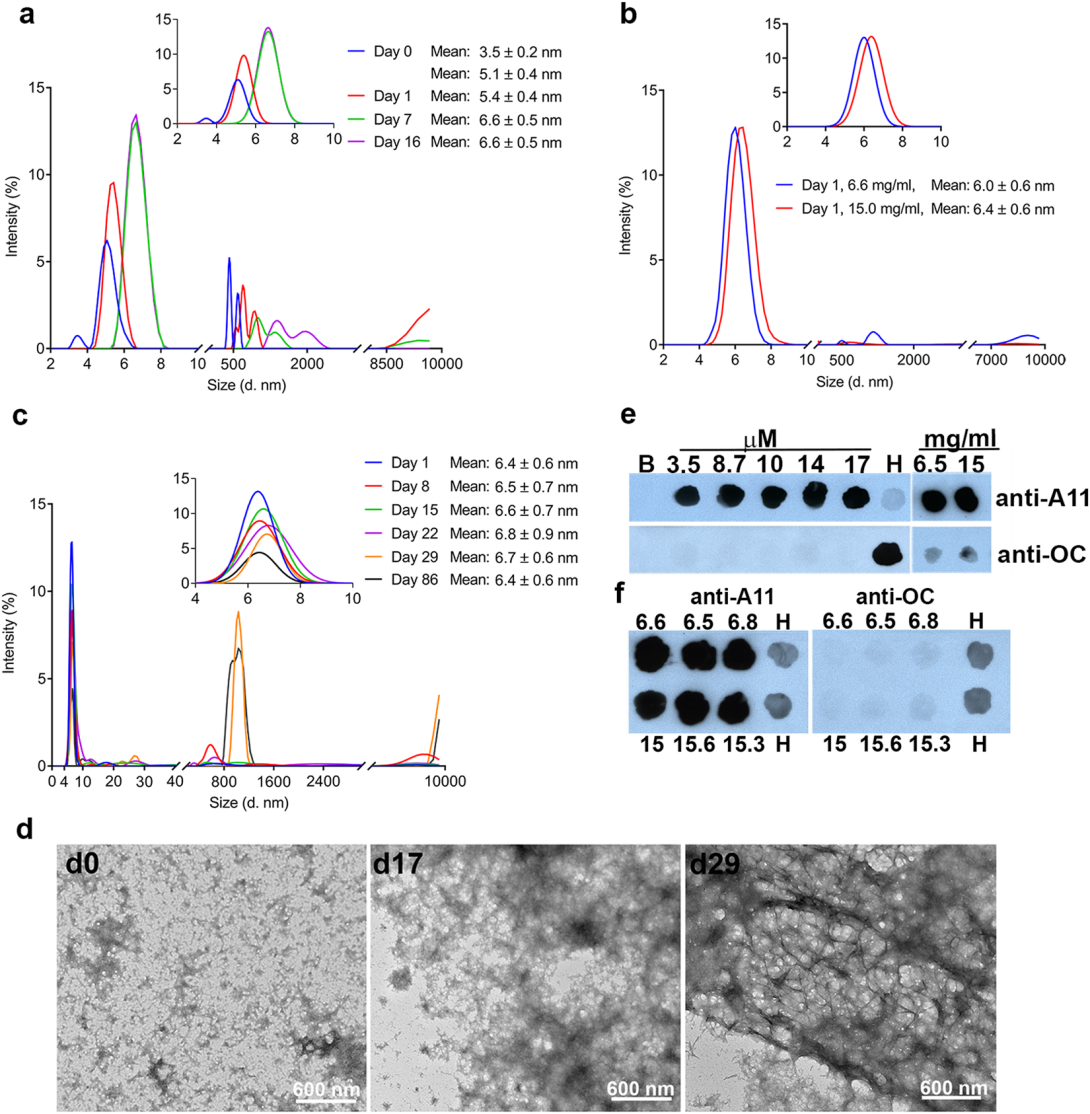
CRES assembles into a stable oligomeric intermediate. (**a**) CRES preparation #4 from Fig. 1 (purple data set) was followed over 16 days by DLS to examine its aggregation properties in solution. Inset, average hydrodynamic radius for the particles between 4–8 nm was calculated from the fitted data and diameter ± SD is reported. Data are representative of n = 3 different protein preparations (see Supplementary Fig. S5). DLS analysis of (**b)** mid- (6.6 mg/ml) (blue) and high (15 mg/ml) (red) concentration CRES in 4 mM potassium phosphate buffer, pH 7.4, 24 hours after purification and concentration. Inset, average diameter ± SD of particles in the 4–8 nm population; and (**c)** high concentration CRES over time (day 1-day 86). Inset, average diameter ± SD of particles in the 4–8 nm population. Data are representative of n = 3 protein preparations (see Supplementary Figs S4 and S5). (**d**) Negative stain TEM of high concentration CRES at day 0, 17, and 29. (**e**) Dot blot analysis of samples from different stock concentrations of CRES (3.5–17 μM) in high salt gel filtration buffer and CRES in 4 mM potassium phosphate buffer, pH 7.4 concentrated to 6.5 mg/ml and 15 mg/ml. All were examined within 24 hours after purification. (**f)** Dot blot analysis of three sets of CRES samples concentrated to mid- (6.5–6.8 mg/ml) and high (15–15.6 mg/ml) concentrations and examined after 45, 60, and 105 days, respectively. For all dot blots 4.4 μg protein was spotted onto nitrocellulose membranes followed by incubation with anti-oligomeric A11 and anti-fibrillar OC antibodies. B, buffer only. H, His-CRES.

To further examine the aggregation propensity of CRES, we monitored its size over time using DLS. When kept at low concentrations of ~0.16–0.23 mg/ml (11–16 μM) in high salt gel filtration buffer, pH 6, similar to samples in Fig. 1b, we observed a time-dependent assembly of CRES monomers (5.1 nm) into larger particles. After 7 days these particles reached 6.6–6.8 nm in diameter which may represent an oligomer (Fig. 2a and Supplementary Fig. S3). This CRES population was stable and detected after 16 days. CRES monomer that underwent ultracentrifugation to remove preexisting aggregates prior to DLS also assembled into a larger particle (6.4 nm) suggesting the larger particle was generated from the monomer (Supplementary Fig. S3). In addition to the transition of monomer into oligomers, CRES aggregates that were present in the starting material also assembled with time into larger structures as indicated by the shift in distribution from a population at 500 nm at day 0 to greater than 2000 nm at day 16 (Fig. 2a and Supplementary Fig. S3). CRES that was buffer exchanged into potassium phosphate buffer, pH 7.4 and concentrated to mid- (6.5–6.8 mg/ml; ~0.4 mM) and high (15–15.6 mg/ml; ~1.0 mM) concentrations, that can occur within cellular organelles such as secretory granules^30^, aggregated more quickly and within 1 day formed oligomers (Fig. 2b and Supplementary Fig. S4). As indicated by the high concentration samples, the CRES oligomer was stable and detected after 86 days incubation; however, at the later times the intensity of this population started to decrease which corresponded with an increased appearance of larger CRES particles including those at 1000 nm and greater than 10000 nm, outside the measurable range for DLS (Fig. 2c and Supplementary Fig. S5). TEM also indicated a change in CRES structure with time. While some granular material was always present, the larger CRES assemblies progressed from oligomeric/protofibril amyloid at day 0 to diffuse matrix at day 17 and then thick fibrils/mesh after approximately 29 days (Fig. 2d). The mid-concentration CRES samples behaved differently during the time course. Although rapidly transitioning to a particle larger than the monomer after initial concentration, this CRES population was less stable and in some samples transitioned back to monomer (5.3–5.4 nm) before abruptly forming an oligomer (6.5 nm–8.8 nm) or larger forms after 29 days (Supplementary Fig. S5). TEM revealed a gradual increase over 29 days in the appearance of larger CRES amyloid assemblies that were similar, although less dense, to those present in the high concentration sample (Supplementary Fig. S5). These results show that CRES amyloid assembly is accelerated by higher protein concentrations and includes the formation of a stable oligomeric intermediate.

We further characterized the amyloid properties of CRES within 1 day of purification using conformation-dependent antibodies in dot blot analysis. The anti-oligomeric amyloid antibody A11, but not the anti-fibrillar amyloid antibody OC, strongly bound to equal μg amounts of CRES taken from stock solutions at different concentrations (3.5–17 µM) suggesting they contained prefibrillar oligomeric but not fibrillar oligomeric forms (Fig. 2e, left panel)^31,32^. The binding of A11 antibody is also consistent with amyloid rich in antiparallel β-sheets^33^. His-CRES (H) was included in the dot blot analyses for comparison since we previously established that following purification from bacterial inclusion bodies it immediately assembled into amyloid following dilution out of 6 M guanidine hydrochloride. In contrast to amyloid assembled from the nondenatured CRES, His-CRES exhibited immunoreactivity to both A11 and OC antibodies, consistent with it forming distinct amyloid forms containing both antiparallel and parallel β-sheets^2^ (Supplementary Fig. S6). Dot blot analysis of all three sets of CRES samples that had been concentrated to the mid- and high concentrations and allowed to age for 29–90 days showed that, despite prolonged incubation and the formation of higher ordered amyloid structures including fibrils, only the anti-oligomeric A11 antibody strongly bound (Fig. 2f). This suggests CRES amyloids from early to advanced assemblies are rich in antiparallel β-sheets and/or possess prefibrillar oligomeric amyloid properties. Although freshly prepared mid- and high concentrations of CRES also strongly bound the anti-A11 antibody, some anti-OC antibody binding was detected (Fig. 2e, right panel). Thus, although high protein concentration facilitates the formation of higher ordered antiparallel β-sheet rich CRES amyloid, there may be additional structural changes that occur with age.

### CRES structural conversion involves α-helix to antiparallel β-sheet transition

**Figure 3:**
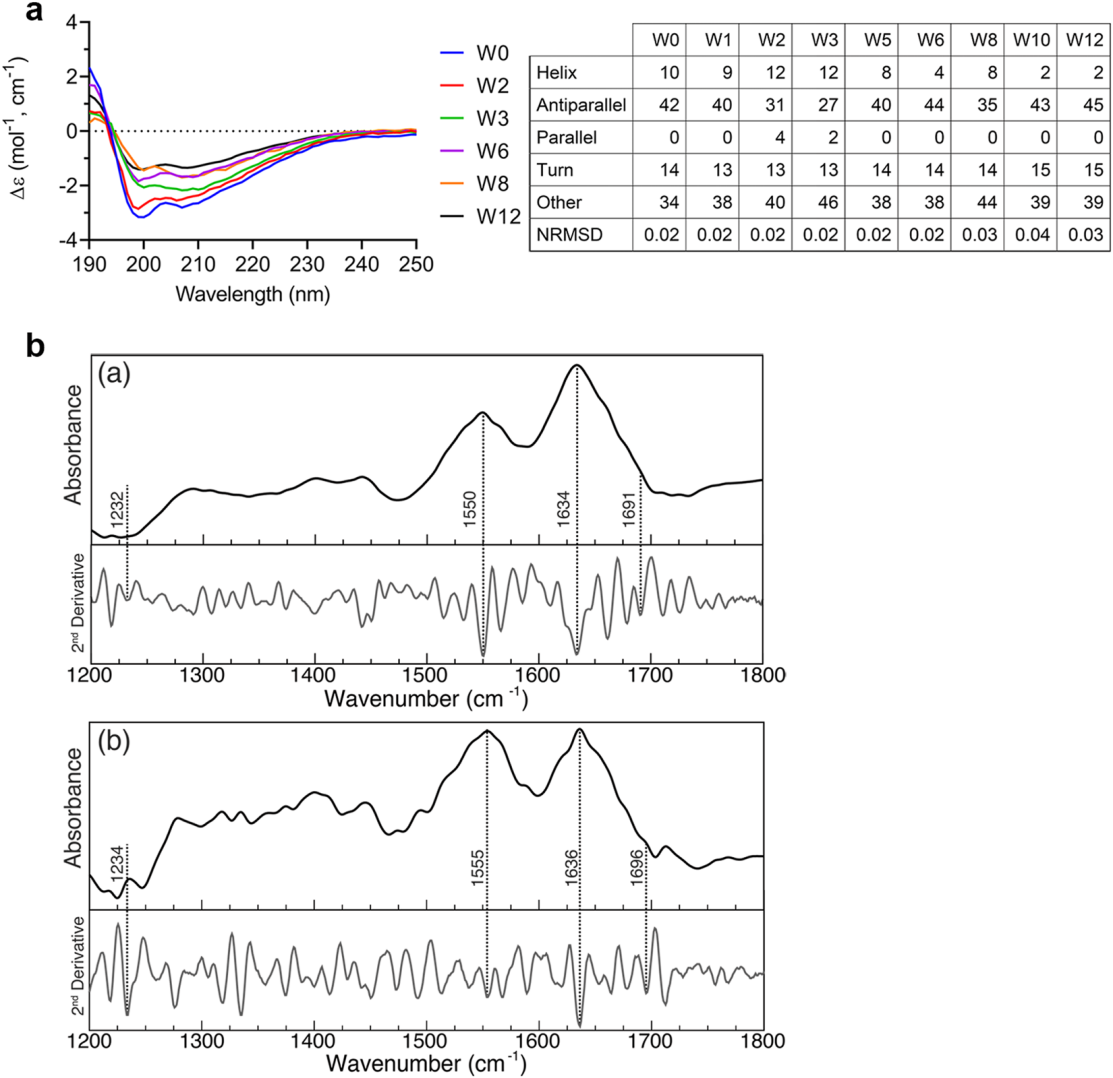
CRES structural conversion involves α-helix to antiparallel β-sheet transition. (**a**) CD was performed approximately once a week for 12 weeks (W0-W12) on the same sample of mid-concentration CRES (6.6 mg/ml) in 4 mM potassium phosphate buffer, pH 7.4. Samples were diluted to 0.17 mg/ml and spectra immediately collected. Spectral curves show experimental data. Secondary structure predictions were done using the BeStSel server^28^. NRMSD, normalized root mean square deviation. (**b)** ATR-FTIR was performed on mid- and high concentration CRES samples after incubation in 4 mM potassium phosphate buffer, pH 7.4 for 2 weeks at 4 °C. Samples were diluted to 0.17–0.21 mg/ml immediately before collection of spectra. (**a**), mid-concentration CRES (6.4 mg/ml); (**b**), high concentration CRES (15 mg/ml). Second derivative spectra are included.

We next used CD to determine if the early oligomeric states of CRES correlated with a change in secondary structure. The same mid- and high concentration samples that were shown by DLS to contain oligomeric forms of CRES were examined within 1 hour after elution from the gel filtration column as a function of concentration and time. Compared to low concentrations of freshly isolated CRES, which contained similar α-helical (18 ± 2%) and antiparallel β-sheet (20 ± 4%) content (Fig. 1d), the mid-concentration CRES preparation in potassium phosphate buffer exhibited a loss of α-helix (10%) and a gain of antiparallel β-sheet content (42%) at time 0 as predicted by the BeStSel server (Fig. 3a). The α-helical content continued to decrease over the time course so that by 12 weeks CRES’ secondary structure was 2% α-helix and 45% antiparallel β-sheet. The small percentage of parallel β-sheets that was present in CRES during the first several weeks of the time course was not detected at 12 weeks (Fig. 3a). A decrease in CRES’ α-helical content over 12 weeks was also predicted by the CONTINLL algorithm; however, compared to the BeStSel analysis, the percentage of α-helix was slightly larger while the percentage of β-sheets was smaller (Supplementary Fig. S7). A comparable loss of α-helix and gain of antiparallel β-sheet content was also observed in the high concentration CRES sample after 12 weeks (Supplementary Fig. S7). Similarly, the small percentage of parallel β-sheet present in the earlier CRES structures disappeared from the aged samples. To confirm our CD results suggesting CRES was rich in antiparallel β-sheets, we performed ATR FTIR with mid- and high concentration CRES samples. The FTIR spectra of CRES showed bands at 1634 and 1636 cm^−1^, 1550 and 1555 cm^−1^, and 1232 and 1234 cm^−1^ in the amide I, II, and III regions, respectively, signifying β-sheets^34^. The bands at 1691 and 1696 cm^−1^ indicated the β-sheets were antiparallel (Fig. 3b)^34^. The CRES FTIR spectra were remarkably similar to FTIR spectra from several antiparallel β-sheet amyloidogenic peptides in cystatin C^35^. Together our results imply that CRES is in motion with conformational fluctuations centered primarily around α-helix to antiparallel β-sheet transitions. In all CRES samples parallel β-sheets were usually observed only in the first few weeks after purification and concentration and then disappeared from the aged samples. These results suggest age-related structural changes may involve parallel β-sheet to antiparallel β-sheet transitions (Fig. 2f).

### Thermal and mechanical stress promote CRES amyloidogenesis

**Figure 4:**
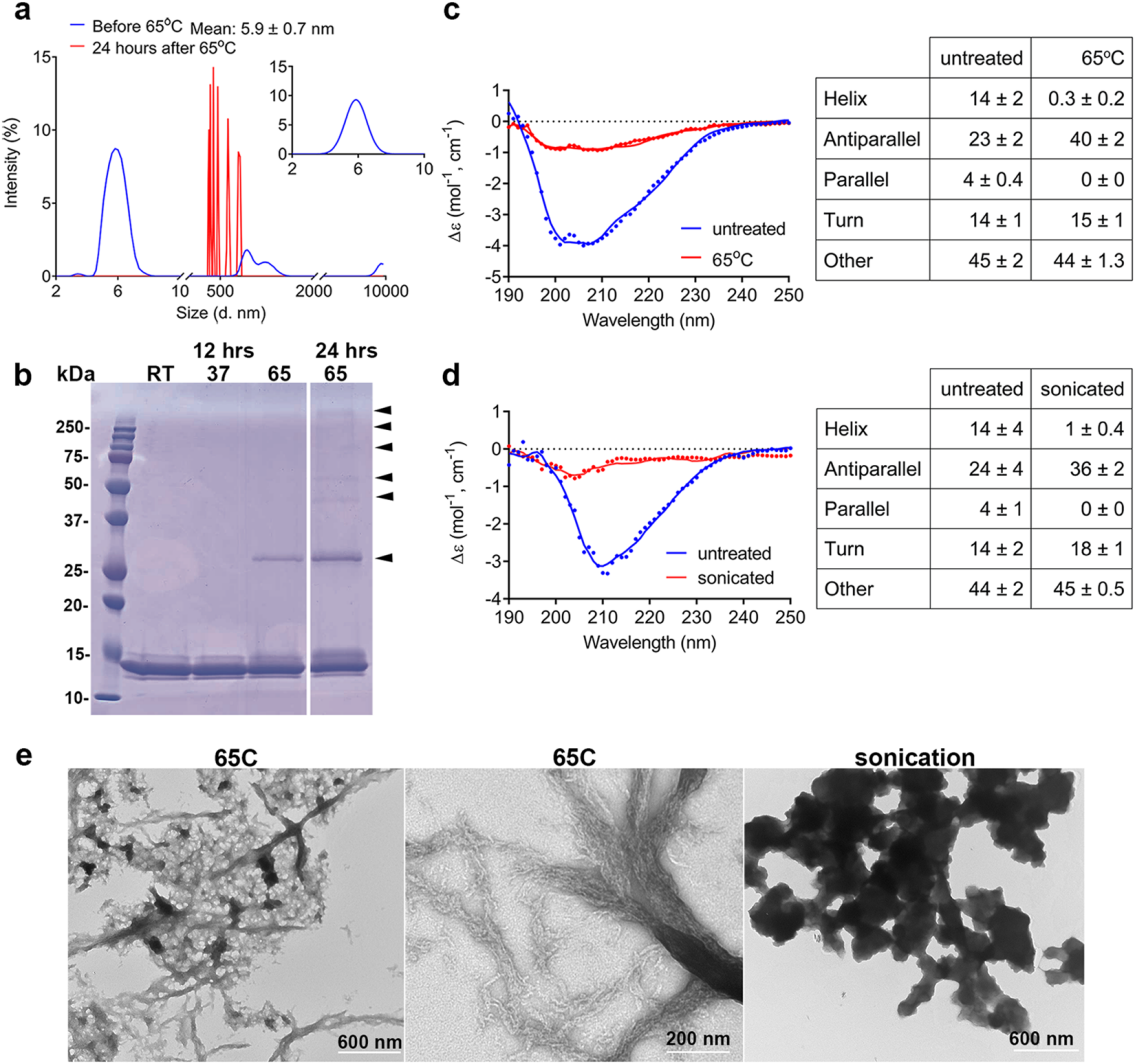
Thermal and mechanical stress promote CRES amyloidogenesis. Freshly prepared CRES was exchanged into 4 mM potassium phosphate buffer, pH 7.4 and analyzed by (**a)** DLS and (**c)** CD before (blue) and after (red) heating at 65 °C for 24 hours. CD spectral curve shows experimental (dotted line) and fitted data (solid line) from a representative experiment. Table shows the mean ± SEM of n = 5 replicates using 3 independent CRES preparations. (**b)** Coomassie Blue stained SDS-PAGE of 0.26 mg/ml CRES in high salt gel filtration buffer incubated at room temperature, 37 °C and 65 °C for 12 hours and 24 hours. (**d)** CRES in 12.5 mM MES, 25 mM HEPES, pH 7.4 was concentrated and sonicated as described in Methods. The sample was diluted to 0.16 mg/ml for CD analysis. The untreated control sample was 0.14 mg/ml CRES in 25 mM MES. CD spectral curve shows experimental (dotted line) and fitted data (solid line) before (blue) and after (red) sonication from a representative experiment. The table shows the mean ± SEM of n = 3 independent CRES preparations. (**e)** Negative stain TEM of CRES samples after 24 hour incubation at 65 °C or sonication. Data are representative of n = 3 experiments.

We also determined whether more profound stressors such as thermal and mechanical stress would cause CRES to assemble into higher ordered amyloid forms and if these amyloids exhibited secondary structures different from those allowed to form naturally over time. Heating at 65 °C for 24 hours resulted in the complete loss of CRES monomer and stable oligomeric forms, as indicated by the absence of CRES particles in the 4–8 nm range, and the formation of larger particles ranging from 300–900 nm in DLS analysis (Fig. 4a). SDS-PAGE of the heated sample showed that, in addition to monomer, SDS-resistant CRES dimers and high molecular weight oligomers were now present, which likely were too large to be detected by DLS (Fig. 4b arrowheads). Indeed, TEM showed CRES had assembled into higher ordered amyloids as indicated by the presence of matrix and protofibrils winding into thick fibrils and large spherical aggregates that densely stained with uranyl acetate (Fig. 4e). Heating also caused a conformational change in CRES secondary structure with a significant loss of α-helix (14 ± 2% to 0.3 ± 0.2%, p < 0.05) and parallel β-sheet (4 ± 0.4 to 0 ± 0), p < 0.05) and an increase in antiparallel β-sheet content (23 ± 2% to 40 ± 2%, p < 0.05) (Fig. 4c).

CRES in MES/HEPES buffer, pH 7.4 was concentrated to approximately 13–17 mg/ml and exposed to mechanical stress by sonication, which resulted in a visible precipitate in the sample. TEM showed CRES had assembled into large spherical aggregates approximately 240 nm in diameter, which appeared to form from large beaded chains, both of which densely stained with uranyl acetate (Fig. 4e). Sonication, like heat, also caused a change in CRES secondary structure with a loss of α-helix (14 ± 4% to 1.0 ± 0.4%, p < 0.05) and parallel β-sheet (4 ± 1 to 0 ± 0, p < 0.05) and a gain of antiparallel β-sheet content (24 ± 4% to 36 ± 2%) (Fig. 4d). Dot blot analysis showed that the sonicated CRES amyloids were highly reactive to the anti-A11 antibody but not the anti-OC antibody suggesting that, like amyloids formed with time, these structures are rich in antiparallel β-sheet content and/or possess prefibrillar oligomeric properties (Supplementary Fig. S8).

### Solid-state NMR confirms the lack of α-helix in CRES amyloid

**Figure 5:**
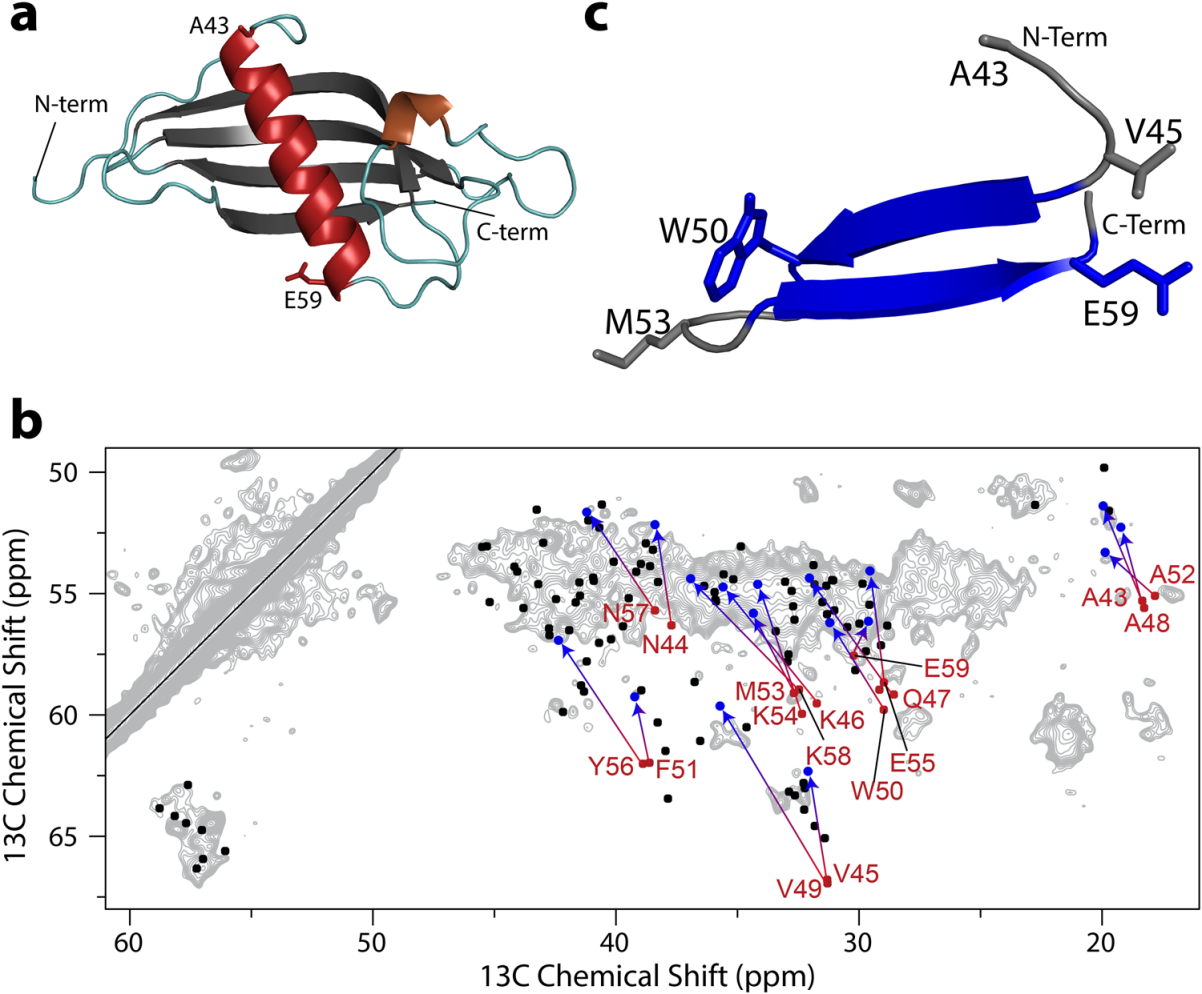
Solid state NMR analysis of ^13^C-^15^N-labeled CRES amyloids. (**a)** Structural model for CRES monomer predicted with SWISS MODEL. (**b)** Chemical shifts for this model were predicted with SHIFTX-2, converted into ^13^C^α^-^13^C^β^ correlation peak lists with FANDAS, and overlaid onto a 2D DARR ^13^C-^13^C correlation spectrum generated from sonicated CRES (gray). The predicted chemical shifts for non-helical sites in the protein agree well with the DARR spectrum (black dots). However, chemical shifts predicted from the α-helix (red) show relatively poor agreement. The agreement between the spectrum and the predicted chemical shifts was improved by converting the secondary structure of the helix into an idealized antiparallel β-hairpin. (**c)**. The movement from the predicted α-helical chemical shifts to the corresponding β-sheet chemical shift for the same site is indicated by the red arrows depicted in (**b)**. The α-helical peak is at the foot, and the predicted β-strand chemical shift is at the head of each arrow. Note that all peaks from the predicted helical region of the protein appear to shift into the strongly-observed β-sheet chemical shift region of the spectrum.

We next acquired a 2D ^13^C-^13^C solid state nuclear magnetic resonance (SSNMR) spectrum of CRES to determine the distribution of secondary structure present after assembly into higher-ordered amyloid forms generated by sonication. The 2D spectrum (gray) revealed a collection of chemical shifts that were largely β-strand or coil (Fig. 5b). In the absence of complete ^13^C chemical shift assignments, we analyzed the data by generating a homology model of CRES using the SWISS-MODEL web server and six cystatin crystal structures. Structural templates were selected from only monomeric (non-domain swapped) cystatin structures. This homology model predicted CRES monomer has the typical cystatin family fold showing an unstructured N-terminus leading into a long α-helix (residues A43 to E59) sitting atop a β-sheet composed of four β-strands (Fig. 5a). We predicted the ^13^C^α^ and ^13^C^β^ chemical shifts of the CRES monomer using SHIFTX-2^36^ and generated peak lists using FANDAS^37^. These predicted chemical shifts were overlaid onto the 2D CRES spectrum with β-strand and coil shifts depicted in black and α-helical shifts depicted in red (Fig. 5b). The predicted α-helical chemical shifts for the model CRES monomer did not agree with the recorded spectra as they do not overlap with the acquired gray signal. The absence of the α-helical shifts in the 2D spectrum indicated that the α-helical structure either unfolded to a random coil or transitioned into a β-strand. To examine the latter hypothesis, we placed residues A43 to E59 into an idealized antiparallel β-hairpin (Fig. 5c) and overlaid their chemical shifts on the 2D spectrum (Fig. 5b) (red dot transitions to blue dot). This moved the residues A43 to E59 so they now overlapped with the CRES spectrum. This suggests in higher-ordered CRES amyloids α-helices have transitioned into β-strands, which supports the CD data in Fig. 4d. The predicted chemical shifts from the four β-strands of the CRES monomer were in good agreement with the recorded spectrum (black shifts overlap with gray signal), confirming the robustness of our model. Based upon the predicted amyloid propensity for these regions of the protein, it may well be the four antiparallel β-strands are largely conserved in the higher-ordered amyloid form of the protein. Taken together, the SSNMR and modeling data help explain the unusually rich antiparallel β-sheet content in CRES amyloid.

### CRES amyloids are not cytotoxic to mammalian cells

**Figure 6:**
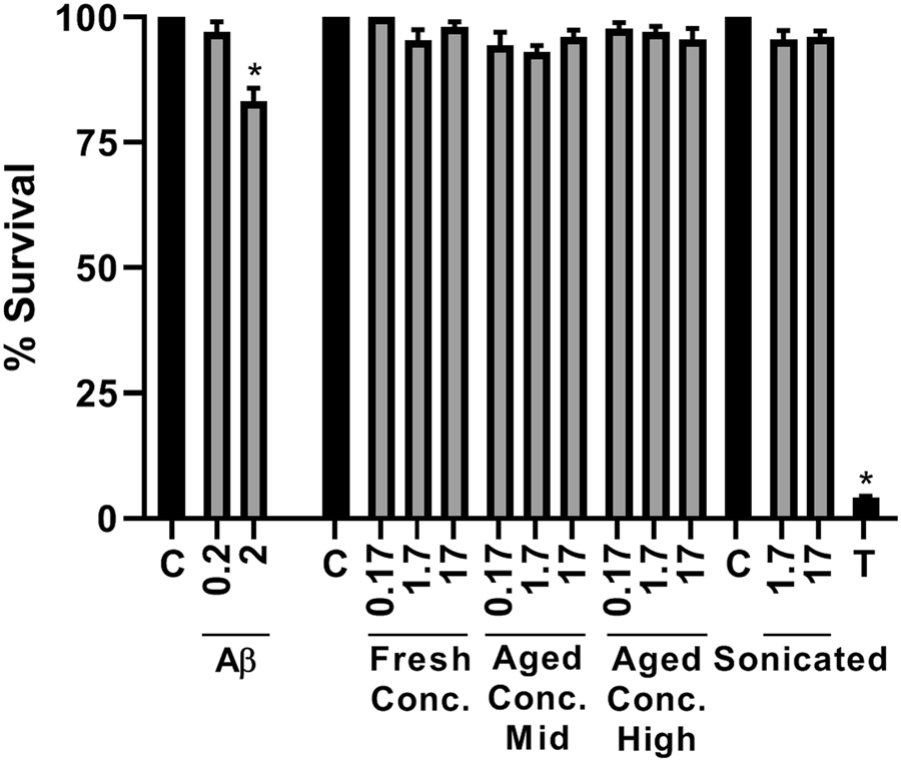
CRES amyloids are not cytotoxic to mammalian cells. Freshly prepared and aged mid- and high concentration CRES was added to mouse DC1 epididymal cells (0.17–17 μM final) in a 96 well plate and incubated for 20 hours at 37 °C, 5% CO_2_ in media without serum. Freshly prepared Aβ_1–40_ added to DC1 epididymal cells (0.2–2 μM final) served as a cytotoxic amyloid control. Cell viability was determined by MTT assay. Cells incubated with freshly prepared mid- and high concentration CRES showed similar MTT values and were pooled. C, control cells incubated with buffer only. T, cells incubated with 1% Triton x-100. All samples were done in duplicate. Data show the mean ± SEM of n = 3–6 biological replicates using 2–5 different CRES preparations. Statistical differences were determined by ANOVA followed by Tukey test. *p ≤ 0.05.

Many amyloids, both functional and pathological, form cytotoxic oligomeric amyloids *in vitro* as an intermediate step during amyloidogenesis. To determine if CRES amyloids are cytotoxic, we examined in cell viability assays several different CRES preparations including freshly prepared CRES concentrated to mid- and high concentrations, and mid- and high-concentration that had been aged from 1–23 weeks. CRES that was sonicated to form spherical amyloid structures was also examined. As shown in Fig. 6, none of the CRES amyloid populations exhibited significant levels of cytotoxicity compared to buffer-only controls after 20 hours of incubation with the DC1 mouse epididymal cells. In contrast, an established cytotoxic amyloid, Aβ1–40^38^ caused a significant reduction in DC1 cell viability compared to buffer control. DLS was performed on the same day CRES amyloid was added to cells in the viability assay to determine if its dilution to 17 μM, the highest concentration examined, caused the oligomer and larger amyloid forms to disassemble. Following dilution the freshly prepared (1–2 days) mid- and high concentrated CRES contained monomer (4.8–5.5 nm) and larger forms ranging from 10–2000 nm (Supplementary Fig. S9). The aged (1–23 weeks) samples maintained more of the larger amyloid assemblies, in addition to generating monomer; however, their ability to generate monomer decreased with increasing age of the sample (Supplementary Fig. S9). The oligomer was not detected in any of the samples suggesting it disassembled to monomer and that high concentrations are required to maintain the metastable state. The sonicated CRES amyloids did not disassemble following dilution (Supplementary Fig. S9). Our results suggest that CRES amyloids from early to advanced forms are not cytotoxic to mammalian cells. If cytotoxic conformers exist they may be present at such low concentration that no effects were detected in the viability assay or they quickly transitioned into noncytotoxic forms, as possibly indicated by the disassembly of the oligomer. The differential response to dilution between fresh and aged samples supports the idea that CRES amyloids may undergo additional conformational changes with age that help stabilize the structure.

### CRES amyloidogenesis is nucleation-dependent

**Figure 7:**
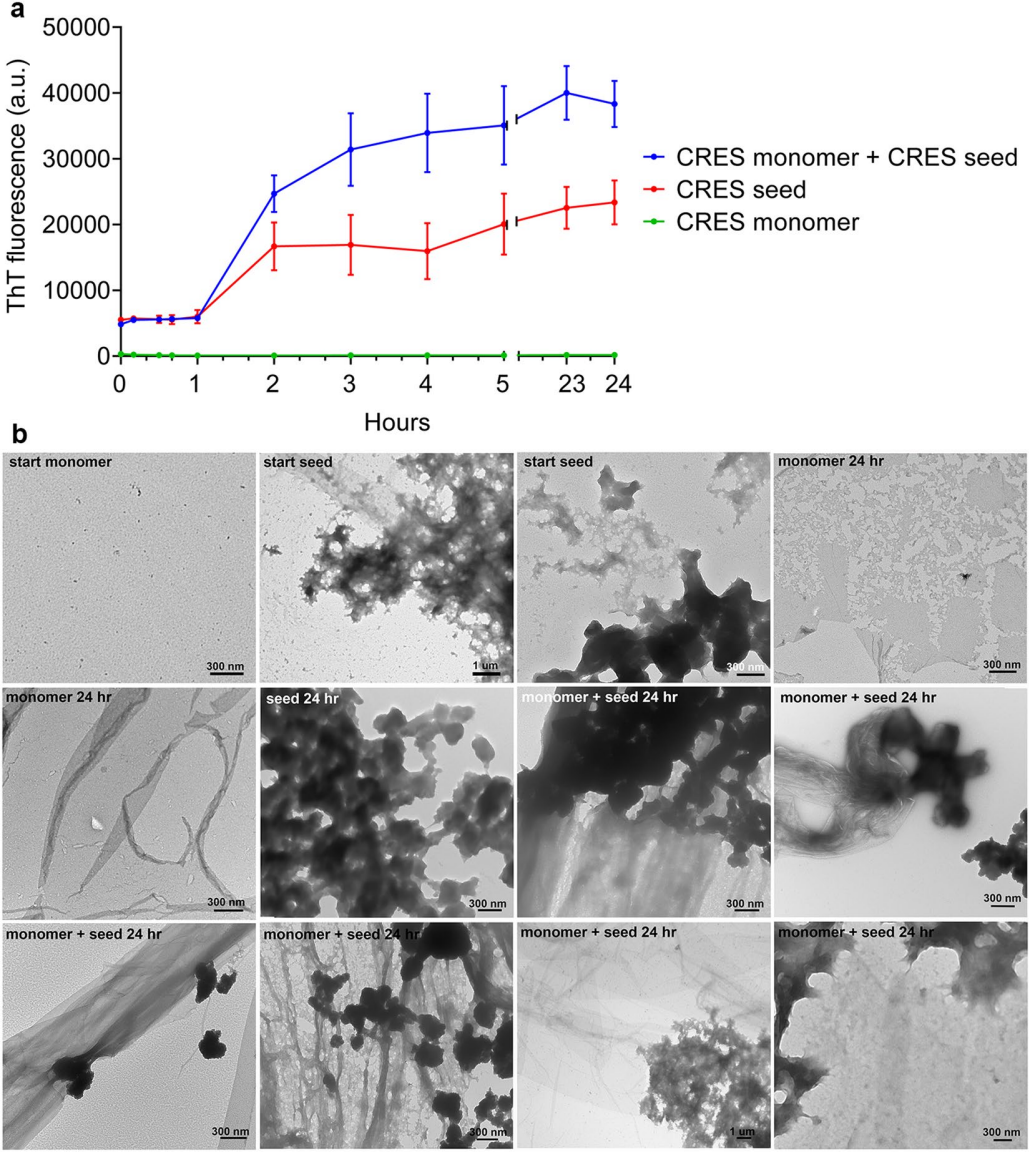
CRES exhibits nucleation-dependent amyloid assembly. (**a)** Thioflavin T fluorescence in 8.7 μM CRES monomer alone, CRES seed, and monomer and seed combined. Monomer to seed molar ratio was 1:1.4. Reactions were done in 20 μM Thioflavin T, 11 mM MES, 20 mM HEPES, 112.5 mM NaCl, and 0.45 mM EDTA, pH 7.4. Fluorescence was measured continuously for 1 hour at 25 °C without shaking and then every hour for 5 hours. Because some evaporation occurred when plates were read overnight, after 5 hours plates were stored covered at RT and read the following day until 24 hours total time. The data show the mean ± SEM from n = 3 experiments using 3 independent CRES preparations. The error bars for the monomer are too small to be detected. (**b)** Negative stain TEM of samples in (**a)** including monomer and seed alone before being added to the ThT reaction (start) and after 24 hours in the ThT reaction (24 hr), and seeded reactions after 24 hours. Monomer, green; seed, red; monomer and seed combined, blue.

Thioflavin T plate assays were performed to determine if CRES amyloid formation proceeds through a nucleated assembly mechanism. Freshly isolated CRES was centrifuged through a 30 kDa filter to remove early aggregates and the filtrate was used within 2 hours as the CRES monomer. CRES that was concentrated, gently sonicated, and allowed to age yielded seeds composed of stacks of protofibrils and dense matrix as well as spherical aggregates, similar to that in Fig. 4 (Fig. 7b start seed). Addition of seeds to monomer caused an increase in ThT fluorescence over that in seeds or monomer alone suggesting these structures served as a template for amyloid assembly (Fig. 7a). TEM analysis of the samples showed that after 24 hours the seeded reactions had an abundance of amyloid films/thick fibrils, often with interacting amyloid matrix or spherical seeds that were not present in the monomer or seed alone (Fig. 7b). In all ThT assays CRES monomer at 8.7 μM was stable and did not assemble into higher ordered amyloid structures over the 24 hour time course as indicated by ThT fluorescence and TEM. Only on a few occasions was film detected in the monomer after 24 hours but these structures were relatively rare (Fig. 7 monomer 24 hr).

### The epididymal amyloid matrix serves as a template for CRES aggregation

**Figure 8:**
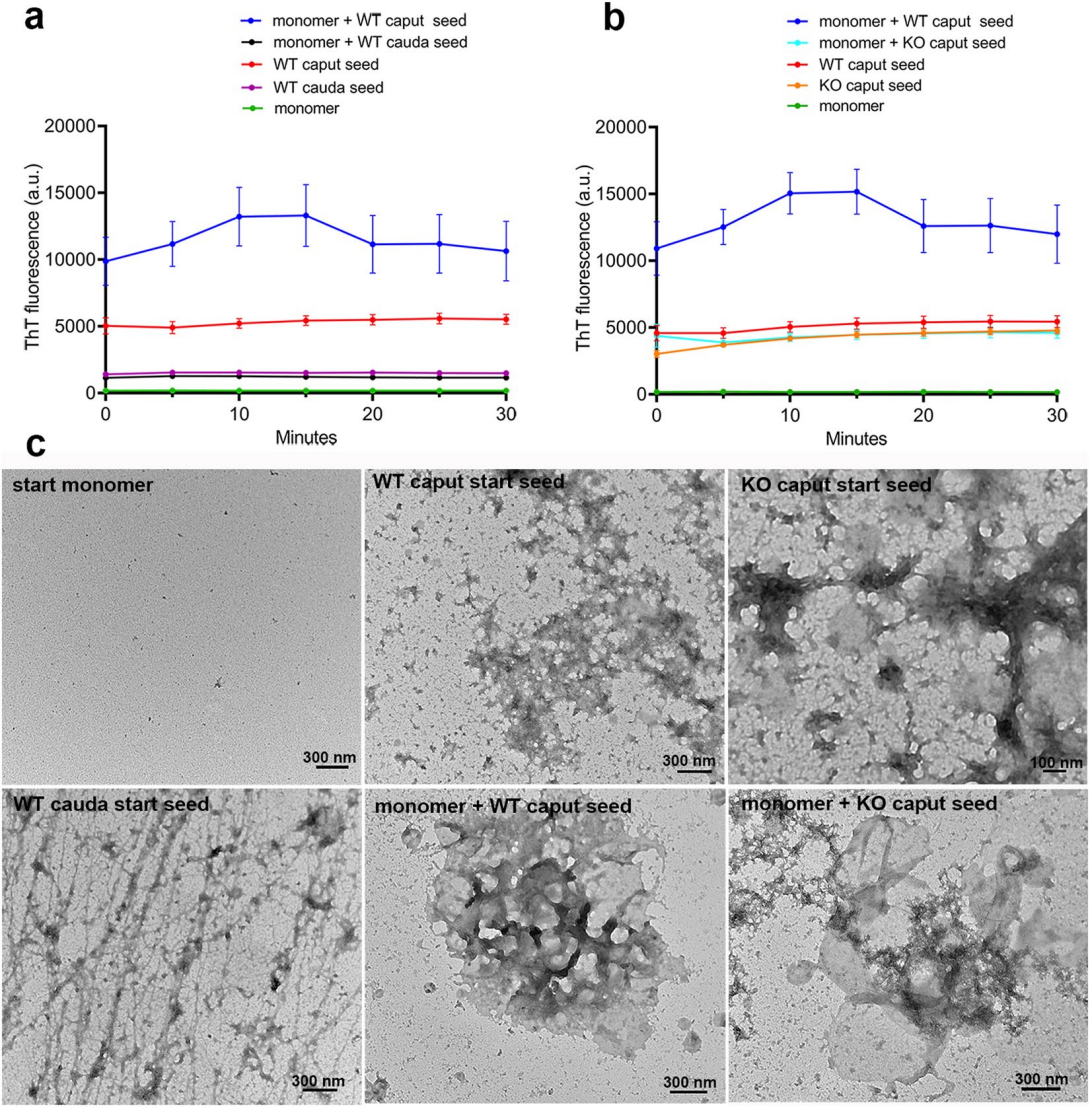
The epididymal amyloid matrix templates CRES amyloid assembly. (**a)** Thioflavin T fluorescence in 4.4 μM CRES monomer, 10 μg caput and cauda epididymal amyloid matrix seed isolated from wildtype (WT) mouse epididymis, and monomer and seed combined. Reactions were done in 20 μM Thioflavin T, 5.5 mM MES, 55–110 mM NaCl, 0.22 mM EDTA, and 0.68X dPBS, pH 7.4. The fluorescence was measured continuously for 30 min at 25 °C without shaking. The data show the mean ± SEM from n = 6 replicates using 3 independent epididymal amyloid matrix isolations. The error bars for monomer, WT cauda seed, and monomer +WT cauda seed are too small to be detected. (**b)** Thioflavin T fluorescence in 4.4 μM CRES monomer, 10 μg caput amyloid matrix seed isolated from WT and CRES KO epididymis, and monomer and seed combined. The ThT data for the WT caput samples are the same as that presented in (**a)** but with one additional replicate. Data are presented in two graphs for clarity. Data show the mean ± SEM from n = 7 replicates using 3 independent epididymal amyloid matrix isolations. The error bars for monomer are too small to be detected. (**c)** Negative stain TEM of structures present in starting monomer, caput and cauda seeds, and seeded reactions from WT and KO samples. Additional TEM images are in Supplementary Fig. S10.

Amyloid matrix isolated from the mouse epididymal lumen was examined to determine if it also would function as a seed for CRES amyloid assembly. Addition of amyloid matrix isolated from the caput but not the cauda epididymal lumen from wildtype (WT) mice caused an immediate increase in ThT fluorescence in the CRES monomer while monomer and caput and cauda seeds alone showed no change over the 30 minute time course (Fig. 8a). The caput epididymal preparation (WT caput start seed) that seeded CRES contained amyloid matrices that resembled the start CRES seeds formed *in vitro* (compare Fig. 8c with Fig. 7b), while the structures in the cauda epididymal preparation that did not seed were distinct and included an abundance of thin fibrils and filaments (Fig. 8c).

We previously demonstrated the loss of CRES from the epididymis in the CRES KO mouse resulted in a noticeably altered amyloid matrix structure^39^. In contrast to WT, caput amyloid matrix from the KO epididymis did not seed CRES monomer with ThT fluorescence levels in the seeded reactions similar to that in WT and KO seeds alone (Fig. 8b). Examination of the caput amyloid seeds alone as well as the seeded reactions by TEM revealed clear differences in the structures from CRES WT and KO samples. The starting KO caput seed was less dense than WT and revealed ball-like structures that were associated with thick ropes (Fig. 8c). Upon further inspection these ball structures were also observed embedded in the WT amyloid matrix. The balls, which ranged in size from 80 nm-500 nm, did not readily stain with uranyl acetate and appeared to be perfectly round unlike the spherical CRES amyloids formed *in vitro* by sonication (Fig. 4e) and those previously found associated with films in endogenous epididymal amyloid matrix^1^. At the end of the ThT experiment the WT and KO caput seeds appeared more dispersed compared to starting material (Supplementary Fig. S10c–f). Whether these changes are a result of dilution in the ThT reaction volume and/or ThT binding have not been determined. The amyloid structures generated in the seeding reactions also were quite different between WT and KO samples. The seeded reaction of WT caput amyloid matrix with CRES monomer contained an abundance of large clusters of interconnected thick fibrils that engulfed both the large and small balls (Fig. 8c and Supplementary Fig. S10g,h). In contrast, the coincubation of KO caput amyloid matrix with CRES monomer, which did not result in seeding, contained disorganized assemblies, some of which looked as though clusters were trying to form (Fig. 8c and Supplementary Fig. S10i,j). Some amyloid matrix structures were also detected that looked similar to WT caput amyloid matrix starting seed suggesting that addition of CRES monomer to the KO amyloid matrix might have recapitulated the WT structure (Fig. 8c and Supplementary Fig. S10k).

## Discussion

Our studies presented herein demonstrate that when purified under nondenaturing conditions, CRES assembles into a metastable antiparallel β-sheet rich oligomeric intermediate that over extended time transitioned into higher-ordered amyloids. The rate of transition from monomer to oligomer was dependent on protein concentration with higher concentrations resulting in a faster transition to the oligomer. In addition to facilitating assembly, high protein concentrations were also necessary to maintain the metastable oligomer since following dilution from millimolar to micromolar concentrations it was no longer detected. We previously demonstrated that, in addition to being a part of the amyloid matrix, CRES monomers, and to a lesser degree dimers and tetramers, are also in the epididymal lumen^40^. Our ability to detect CRES dimers and tetramers, however, required chemical crosslinking suggesting the stability of these forms *in vivo*, like oligomers formed *in vitro*, may be concentration-dependent^40^. Together, our results suggest the assembly states of CRES *in vitro* mimic that which occurs within the epididymal lumen. Endogenous stable tetramer/oligomers have also been described for other amyloidogenic precursors including α-synuclein and TasA^41,42^. Although studies with denatured protein suggested α-synuclein is a natively unfolded protein that quickly assembles into a parallel β-sheet rich amyloid, recent studies of a nondenatured protein showed that it exists *in vivo* as a metastable α-helical tetramer that resists amyloid formation^41^. Further, similar to CRES, optimal detection of the α-synuclein tetramer *in vivo* required chemical crosslinkers in the presence of high protein concentrations to prevent its destabilization^43^. When purified under nondenaturing conditions, the functional bacterial amyloid precursor TasA, which contributes to biofilm formation in *Bacillus subtilis* much like the well-characterized Curli proteins in *E. coli*, was a stable α-helical oligomer that resisted aggregation^42^. Together these studies suggest that early metastable oligomers may be a common building block mechanism for assembly of some functional amyloids and raise the intriguing possibility the α-synuclein oligomer could also be a functional structure^44^.

In addition to concentration affecting CRES amyloid assembly, aging also resulted in additional conformation changes that generated an amyloid structure more resistant to reversal and/or that disassembled differently than a freshly aggregated protein. During amyloid assembly DLS showed the stable CRES oligomer gradually reduced in intensity with time suggesting it served as a building block for larger forms. However, following dilution the disassembly of CRES followed a different pathway and the age of the aggregate dictated the reversal process. Mid- and high concentration CRES amyloids generated monomers during their disassembly instead of oligomers; however, with increased age the ability to generate monomers decreased. Other functional amyloids such as the repeat domain of Pmel17 also release monomer rather than oligomer during disassembly and this has been proposed to be a means to protect the cell from potential oligomer-related cytotoxicities^45,46^.

The transition of CRES into higher-ordered amyloids correlated with a change in secondary structure with a loss of α-helix and a gain of β-sheets. This is supported by our early SSNMR studies of sonicated CRES which indicated that, based on models, at the atomic level CRES amyloid lacked α-helices and was rich in β-sheets, turn, and coil geometries. Although the transition of CRES secondary structure from α-helix to β-sheets is typical of conformational changes that occur in both functional and pathological amyloid precursors, CRES is distinct in that CD and FTIR analysis predicted it assembles into an antiparallel β-sheet rich amyloid rather than the more common parallel β-sheet form. The few amyloids that have been shown to consist of antiparallel β-sheets include a mutant form of Aβ (Iowa mutation), which formed oligomers that were highly cytotoxic to mammalian cells, a cytotoxic oligomer-forming segment of αB-crystallin, which crystallized as an antiparallel β-barrel oligomer called cylindrin, and several amyloidogenic peptides from cystatin C^35,47,48^.

Although establishing the higher-ordered CRES amyloids as being antiparallel β-sheet rich awaits confirmation by SSNMR, CRES in its early and advanced amyloid forms, like cylindrin and other amyloid oligomers, strongly bound the anti-oligomeric A11 antibody but not the anti-fibrillar OC antibody. The exact epitope that the anti-A11 antibody binds to is not known but it recognizes an element that is common to many different oligomeric amyloids classified as prefibrillar oligomers that are thought to be antiparallel β-sheet rich structures that follow a different aggregation pathway than anti-OC reactive fibrillar oligomers which eventually yield fibrils^32^. The significance of these two pathways is unclear but studies have shown antiparallel β-sheet oligomers of functional and pathological amyloids to be cytotoxic to mammalian cells, suggesting that, like parallel β-sheet oligomers, they may contribute to disease^33,47–49^. Unlike other antiparallel β-sheet rich amyloids, none of the CRES amyloids tested showed significant cytotoxicity against mouse distal caput DC1 epididymal cells in culture. This suggests CRES amyloid forms may be distinct from others described. Although its destabilization following dilution prevented us from determining the cytotoxicity of the CRES oligomer, if it is indeed cytotoxic its disassembly could be a mechanism to prevent accumulation of cytotoxic forms.

In support the anti-oligomeric A11 reactive CRES amyloids are functional rather than pathological are our previous studies showing the endogenous CRES-containing amyloid matrix in the caput epididymis is also highly reactive to the anti-oligomeric A11 antibody^39^. This suggests a structure rich in antiparallel β-sheet and/or prefibrillar oligomeric amyloids is a normal component of the mouse epididymal lumen in the proximal part of the epididymis. These results, taken together with our previous TEM analyses^1^, suggest the caput epididymal amyloid matrix is a structurally heterogeneous and perhaps immature amyloid structure while the cauda amyloid matrix is less heterogeneous and a more advanced amyloid matrix. The caput amyloid matrix structures also functioned as seeds for the assembly of CRES amyloid *in vitro* while the cauda structures did not. Studies of other A11 reactive amyloid oligomers have shown these structures to be the species capable of cross-seeding with other amyloidogenic precursors^50^. Considering the epididymal amyloid matrix consists of multiple cystatins, the A11 reactive forms may allow CRES to heterooligomerize with other CRES subgroup members and cystatin C, and possibly with unrelated amyloid precursors which may be integral for amyloid matrix function. These heterotypic interactions could also be a mechanism to regulate the assembly of the functional amyloid matrix structure, not unlike in *E. coli* where interactions between Curli family members control formation of the functional amyloid that contributes to biofilm formation^51^.

Taken together, the studies presented herein reveal that nondenatured CRES exhibits many of the properties of other functional amyloids including nucleation-dependent assembly, the formation of an anti-A11 immunoreactive oligomeric intermediate and α-helix to β-sheet secondary structure transition. However, CRES is also distinct in that it forms antiparallel β-sheet rich amyloids which, under the conditions tested, were not cytotoxic to mammalian cells; moreover, similar amyloid conformers seem to be present *in vivo*. Indeed, while most studies have focused on amyloid fibrils as the physiological form, the heterogeneity of amyloid structures in the epididymal lumen including films, spheres, loose and dense matrix, protofibrils, filaments, and fibrils, argues that conformations in addition to fibrils likely perform biological roles^20,52^. Further studies of CRES structure at the atomic level will help reveal potential mechanism(s) that define functional amyloids from the pathological forms.

## Materials and Methods

### Animals

*Cst8* 129SvEv/B6 gene knockout and wildtype mice were bred in house. Mice were maintained under a constant 12 h light/12 h dark cycle with food and water *ad libitum*. All animal studies were conducted in accordance with the NIH Guidelines for the Care and Use of Experimental Animals using a protocol approved by the Texas Tech University Health Sciences Center Institutional Animal Care and Use Committee.

### Expression and purification of GST-free CRES

Full-length mouse CRES (*Cst8*), Swiss-Prot accession number P32766, lacking the signal sequence (residues 1–19) and containing residues 20–142 was modified with a cysteine 48 to alanine change (C48A) to prevent inappropriate disulfide bond formation during protein expression and was cloned into pUC57 (Genewiz, South Plains, NJ). CRES C48A was then cloned into the pGEX-cs vector as a glutathione S-transferase (GST) fusion protein and transformed into Origami B *E. coli* for expression. *E. coli* were grown in M9 medium containing D-glucose (4 g/l) and ammonium chloride (1 g/l) as sole sources of carbon and nitrogen, respectively. The expression of GST-CRES was induced by 0.4 mM IPTG at OD _600_ 0.8–1.0 and growth continued at 12 °C for 20–22 hours in a shaking incubator. The cells were pelleted at 4785 × g, 4 °C for 20 minutes and resuspended in 100 ml lysis buffer (250 mM NaCl, 25 mM Tris, and 1 mM EDTA, pH 8.0)/1 L cell pellet. The cells were broken using a microfluidizer processor (Microfluidics, Co., Westwood, MA) with a pressure between 15000 psi–20000 psi. Soluble proteins containing GST-tagged CRES were separated from the cell debris by spinning at 34500 × g, 4 °C for 45 minutes and then loaded on to a glutathione agarose column (Pierce Glutathione Superflow Agarose (product # 25237, Thermo Scientific, Rockford, IL). The elution was conducted as recommended by the manufacturer except that GST-tagged CRES was separated from the glutathione beads using 0.5% w/v reduced L- glutathione (Sigma-Aldrich, St. Louis, MO) in the lysis buffer, adjusted to pH 7.4. The GST tag was removed using TEV protease at a ratio of 14 mg GST-tagged CRES to 1 mg TEV at 4 °C for at least 12 hours. The NaCl concentration was then diluted to 41.6 mM using 25 mM MES, 1 mM EDTA, pH 6.0 before loading on to a SP Sepharose Fast Flow column (GE Healthcare, Chicago, IL). To separate GST-free CRES and uncut GST-tagged CRES from TEV and GST proteins, a NaCl gradient built from buffers A (50 mM NaCl, 25 mM MES, 1 mM EDTA, pH 6.0) and B (1 M NaCl, 25 mM MES, 1 mM EDTA, pH 6.0) was used to elute CRES from the SP column using an NGC Chromatography system (Bio-Rad, Hercules, CA). Finally, monomer was obtained by passing CRES through a Superdex 75 pg gel filtration column (GE Healthcare, Chicago, IL) in gel filtration buffer (250 mM NaCl, 25 mM MES, 1 mM EDTA, pH 6.0). Immediately after elution CRES was diluted to 0.127 mg/ml or lower for storage at 4 °C. Protein concentration was determined by using a Nanodrop Lite spectrophotometer (ThermoScientific, Rockford, IL) with an extinction coefficient of 16960 M^−1^cm^−1^ calculated based on CRES residues 20V-142V and a six residue N-terminal linker, GAMAHM.

### Expression and purification of His-CRES

His-tagged mouse CRES protein was expressed in *E. coli* and isolated from inclusion bodies in 6 M guanidine-Cl, 25 mM MES (2-(4-morpholino)-ethane sulfonic acid) buffer, pH 5 as previously described^53^.

### CRES buffer exchange and concentration

Approximately 6–7 milligrams of tagless CRES in gel filtration buffer were exchanged with 4 mM potassium phosphate buffer, pH 7.4 using an Amicon Ultra-15 10 K centrifugal filter (Millipore, Burlington, MA). CRES was then concentrated to 6.5–6.8 mg/ml (mid-concentration) or 15–15.6 mg/ml (high concentration) using an Amicon Ultra-0.5 10 K centrifugal filter. These samples were used for DLS, CD, dot blot, and cell viability assays as described below.

### Dynamic light scattering

CRES freshly eluted off the gel filtration column at concentrations ranging from 0.17–0.24 mg/ml was examined by DLS within 2 hours of elution. Other samples were examined after buffer exchange to remove the NaCl as described above and were examined without dilution. 100 µl of each sample was placed in a ZEN0040 micro UV-Cuvette (Brand GMBH + CO KG) and analyzed at 25 °C using a Zetasizer Nano ZS (Red) (ZEN3600, Malvern Instrument) equipped with a 633 nm red laser and 173° scattering angle. “Size” and “Protein” were selected as the measurement type and the material type, respectively. For each sample, the measurement was repeated at least 3 times. Each time, 15 scans were acquired, and each scan lasted for 15 seconds. After the diffusion of a particle moving under Brownian motion was measured, the Zetasizer software version 7.11 converted the diffusion to a size and generated size-intensity distributions using Stokes-Einstein relationship with a refractive index of 1.33 nD and dynamic viscosity of 0.8872 cP. Gaussian fitting was then used to extract the size mean. The analysis was done using the 300 size classes with the lower and upper size limits set to 0.4 nm and 10000 nm, respectively.

### Thioflavin T seeding assays

CRES amyloid formation was monitored in 96 well black flat bottom plates (Corning, NY) using a TECAN Infinite M1000 PRO microplate reader (Tecan, Baldwin Park, CA) in the fluorescence top reading mode. The excitation and emission wavelengths were 440 nm ± 5 and 485 nm ± 10, respectively. ThT fluorescence was determined by averaging at least 10 reads and subtracting the ThT blank. To generate the CRES monomer, 15 ml of CRES stored at approximately 0.13 mg/ml at 4 °C in gel filtration buffer for 1–5 days was centrifuged using an Amicon Ultra-15 30 K filter at 2500 × g for 10 minutes, 4 °C. The filtrate was collected and concentrated back to 0.29 mg/ml using an Amicon Ultra-15, 10 K and centrifugation at 2500 × g, 4 °C. This sample was kept on ice and used within 2 hours after it was generated.

To generate the CRES seed, 2.3 mg CRES in gel filtration buffer was buffer exchanged with 25 mM MES, pH 6.0 until the NaCl concentration was below 1 mM. The pH was adjusted to pH 7.4 with 50 mM HEPES, pH 8.0 and the sample further concentrated to 14 mg/ml to 18 mg/ml using an Amicon Ultra-0.5 10 K filter. Eighty μl was removed for sonication at 10 pulses 3X at 20% duty cycle; 10 pulses 3X at 30% duty cycle after which half of the sample (40 μl) was further sonicated at 10 pulses 3X at 50% duty cycle. This sample was used as a seed within 3–11 days after sonication. Seeds were diluted1:4 with 12.5 mM MES, 25 mM HEPES, pH 7.4 buffer and 5 uL was added into the 100 μl ThT reaction containing 8.7 μM CRES monomer and 20 μM ThT (Sigma Chemical Co, St. Louis). For seeding assays with the epididymal amyloid matrix, 10 μg of amyloid matrix in dPBS was added to 4.4 μM CRES monomer and 20 μM ThT.

### Dot blot analysis

Approximately 4 μgs of CRES in gel filtration buffer or His-CRES in 25 mM MES, pH 5 were spotted onto nitrocellulose membrane (Biotrace, Pall Corp, Ann Arbor, MI, USA) and incubated with anti-oligomer A11 antibody and anti- fibrillar OC antibody (Millipore, Billeria, MA, USA) at 1:5000 in 3% milk/TBST overnight at 4 °C as described previously^40^.

### Circular dichroism

CRES was buffer exchanged with 4 mM potassium phosphate buffer, pH 7.4 or 2.5 mM MES/5 mM HEPES pH 7.4 buffer using a PD10 column (Sephadex G-25M, GE Healthcare) and protein concentration was determined by Nanodrop using the extinction coefficient for CRES of 16960 M^−1^cm^−1^. His-CRES was buffer exchanged with 2.5 mM MES pH 5. Proteins were diluted to 11–21 μM with their corresponding buffers and circular dichroism was performed using a J-815 CD spectrophotometer (JASCO Co., Easton, MD). Spectra were acquired at 22 °C in the 190–260 nm spectral range at an acquisition rate of 1 nm/sec and a data pitch of 0.1 nm. Six CD spectra of each sample were averaged to calculate the final CD data. CD spectra were also measured for each buffer and were subtracted from the respective protein containing sample spectra. The secondary structure content of the samples was predicted from the spectral data using the BeStSel web server at http://bestsel.elte.hu/index.php with “input units” of measured ellipticity (mdeg), 129 residues of CRES, and pathlength of 0.1 cm. Spectra were also analyzed using the CONTINLL algorithm (protein reference data sets set 4 and SMP180) with the DichroWeb server^29,54–58^. Means ± SEM were calculated from the predicted secondary structures of n = 3–5 independent CRES preparations and statistical differences were determined by t-test.

### Attenuated total reflectance Fourier-transform infrared spectroscopy (ATF FTIR)

Mid- and high concentration CRES samples, 6.4 mg/ml and 15 mg/ml, respectively, in 4 mM potassium phosphate buffer, pH 7.4 were incubated for 2 weeks at 4 °C. An aliquot was removed from each and diluted to 0.17–0.21 mg/ml with 4 mM potassium phosphate buffer, pH 7.4. Infrared spectra were obtained at a resolution of 4 cm^−1^ using a FTIR spectrometer equipped with a BioATRII unit for measurement of liquid samples (Bruker Optics, Billerica, MA). Absorbance band maxima were determined from the minima in the second derivative which was determined using the Grace software http://plasma-gate.weizmann.ac.il/Grace/.

### MAS solid-state NMR

Uniformly^13^C- and ^15^N- labeled CRES was expressed in *E. coli* grown in M9 medium containing ^13^C-D-glucose (4 g/l) and ^15^N-amonium chloride (2 g/l) (Cambridge Isotope Labs, Andover, MA) and supplemented with ^13^C-^15^N labeled Cell Tone Base powder (1 g/l) (Cambridge Isotope Labs, Andover, MA). CRES purification was performed as described above. Seven milligrams of ^13^C, ^15^N- labeled CRES (29 mg/ml in gel filtration buffer) was sonicated 60 pulses 5X at 20% duty, 60 pulses 5X at 30% duty and centrifuged at 16000 × g for 2 min 4 °C to pellet the insoluble material. To recover sufficient CRES amyloid for analysis, the resulting supernatant was sonicated 60 pulses 5X at 30% duty, combined with the previous pellet, and 50 mM HEPES, 100 mM NaCl, pH 8 added to adjust pH to 7.4. The sample was centrifuged at 16000 × g for 2 min 4 °C to generate pellet 1. The supernatant was sonicated 60 pulses 10 × 30% duty and centrifuged at 16000 × g to generate pellet 2. The pellets were combined to yield 4.4 mg ^13^C-^15^N –labeled CRES amyloid for SSNMR analysis.

The^13^C-^15^N -CRES amyloid sample was packed into a 3.2 mm pencil rotor (Agilent Technologies, Santa Clara, CA and Loveland, CO). SSNMR experiments were carried out on a 600 MHz Agilent DD2 three-channel spectrometer equipped with an HCN Balun probe (Agilent Technologies). The magic-angle spinning (MAS) rate and the sample temperature were maintained at 13.333 ± 2 kHz and at 4 ± 2 °C, respectively. ^13^C chemical shifts were externally referenced using an adamantine sample. The downfield signal of adamantine was set to 40.48 ppm on the DSS scale. The two-dimensional DARR spectrum^59^ was acquired employing 1 ms of ramped cross polarization^60^ and radio frequency fields of 60 kHz on ^13^C and 73 kHz on ^1^H. During cross polarization a 15% tangent ramp was applied to ^13^C. Data were acquired using 8 ms of ^13^C chemical shift evolution, 25 ms of DARR mixing, and 20 ms of directly-detected acquisition. 80 kHz of SPINAL-64^61^ proton decoupling was applied during both indirect and direct chemical shift evolution periods. The spectrum was processed using NMRPipe^62^ with an apodization of 30 Hz Gaussian line broadening in both dimensions prior to Fourier transformation. The data were analyzed in NMRFAM-SPARKY^63,64^. The CRES homology model was generated by SWISS MODEL^65,66^ using six monomeric cystatin crystal structures PDB IDs: 4N6L (human cystatin E/M)^67^, 1A67(chicken egg white cystatin)^68^, 1CEW (chicken egg white cystatin)^69^, 1A90 (chicken egg white cystatin)^68^, 3GAX (human cystatin C)^70^, 1RN7 (human cystatin D)^71^ and chemical shifts were predicted by SHIFTX-2^36^ and converted into peak lists using FANDAS^37^.

### SDS-PAGE

Ten μgs of CRES were separated on a precast 15% Tris-glycine SDS-PAGE gel (Criterion, Bio-Rad, Hercules, CA) under reducing conditions and stained with 0.25% Coomassie Blue for 20 min followed by destaining in acetic acid/methanol.

### Cell viability assay

Mouse distal caput epididymal DC1 cells^72^ were plated at a concentration of 3 × 10^4^ cells/well in a 96 well plate (Falcon, Corning, NY) in 100 µl IMDM supplemented with 10% fetal bovine serum (Atlanta Biologicals, Flowery Branch, GA), 1 mM sodium pyruvate, 0.1 mM non-essential amino acid, 4mM L-glutamine, 4.2 U/ml penicillin, 4.2 µg/ml streptomycin, and 0.001 µM 5 alpha-DHT (Sigma Chemical, St. Louis, MO). All cell culture reagents were from Gibco/ThermoFisher Scientific (Waltham, MA) unless stated otherwise. After 24 hours at 37 °C, 5% CO_2_, the media was removed and replaced with media without serum containing 0.17–17 μM CRES (mid- and high concentration, prepared as described above) in 4 mM potassium phosphate buffer, pH 7.4, sonicated CRES in 12.5 mM MES/25 mM HEPES, pH 7.4, or the corresponding buffer alone as a control. All samples were done in duplicate. Serum was excluded from the media since CRES amyloids *in vivo* would not be exposed to serum proteins as a result of the blood-epididymal barrier. Additional controls included cells exposed to 1% Triton-X-100 as a killing control and cells incubated with 0.2–2 μM Aβ_1–40_. Aβ_1–40_ peptide (HCl salt) (0.5 mg) purchased from rPeptide (Watkinsville, GA) was resuspended in 50 μl 100% DMSO and aliquots were stored at −20 °C. Immediately before use an aliquot was thawed and a 500 μM stock prepared by adding dPBS. At the Aβ concentrations examined and in the corresponding buffer control, the final DMSO in the samples was 2.2%. After 20 hours, the media and protein/peptide were removed and 100 µl media without serum and phenol red was added to each well. Cell viability was determined using the *in vitro* toxicology assay kit (Sigma Chemical, St. Louis, MO) following the manufacturer’s instructions. Briefly MTT (3-[4,5-dimethylthiazol-2-yl]-2,5 diphenyl tetrazolium bromide) was dissolved into 3 ml media without serum and phenol red and 10 µl was added to each well. The samples were incubated for 4 hours at 37 °C, 5% CO_2_ after which 100 µl solubilization solution was added and the cell suspension mixed every 20 min for 1 hour. The plate was read using a TECAN Infinite M1000 PRO microplate reader (Tecan, Baldwin Park, CA) and the absorbance at 650 nm was subtracted from that at 570 nm. Experiments were repeated 3–6 times using 2–5 independent CRES protein preparations and statistical analysis was performed by ANOVA followed by a Tukey post-test.

### Isolation of epididymal amyloid matrix

Caput and cauda epididymal amyloid matrix were isolated from 29–31 week old age-matched CRES wildtype (WT) and knockout (KO) mice as previously described^1,2^. Two mice of each genotype were used for each isolation. Proteins were quantitated using the BCA assay (ThermoScientific, Waltham, MA).

### Negative stain TEM

Recombinant proteins and isolated epididymal amyloid matrix were spotted on to formvar/carbon coated 200 mesh nickel grids (Ted Pella, Redding, CA) as previously described^1^.

## Supplementary information

Supplementary Information

## Data Availability

All data generated or analyzed during the current study are included in this published article (and its Supplementary Information files).
