# Supplementary material for: The Functional Mammalian CRES (Cystatin-Related Epididymal Spermatogenic) Amyloid is Antiparallel β-Sheet Rich and Forms a Metastable Oligomer During Assembly
Source: Sci Rep. 2019 Jun 25;9:9210. doi: 10.1038/s41598-019-45545-w (PMC6593142; doi:10.1038/s41598-019-45545-w)
Supplement: Supplementary file 1 — Supplementary Information [file 41598_2019_45545_MOESM1_ESM.pdf]

The Functional Mammalian CRES (Cystatin-Related Epididymal Spermatogenic) Amyloid is Antiparallel β-Sheet Rich and Forms a Metastable Oligomer During Assembly

Hoa Quynh Do[1], Aveline Hewetson[1], Caitlyn Myers[1], Nazmul H. Khan[4],

Mary Catherine Hastert[3], Faraz M. Hasini[2], Michael P. Latham[4], Benjamin J. Wylie[4],

R. Bryan Sutton[2], Gail A. Cornwall[1]*

[1]Department of Cell Biology and Biochemistry, Texas Tech University Health Sciences Center, Lubbock, TX.

[2]Department of Cell Physiology and Molecular Biophysics, Texas Tech University Health Sciences Center, Lubbock, TX. [3]College of Arts and Sciences Microscopy, Texas Tech University, Lubbock, TX. [4]Department of Chemistry and Biochemistry, Texas Tech University, Lubbock, TX.

Keywords: functional amyloid, epididymis, antiparallel, β-sheet, oligomer, mouse, reproduction

***Correspondence:** Gail A. Cornwall, Ph.D., Department of Cell Biology and Biochemistry, Texas Tech University Health Sciences Center, 3601 4th Street, Lubbock, TX 79430, gail.cornwall@ttuhsc.edu

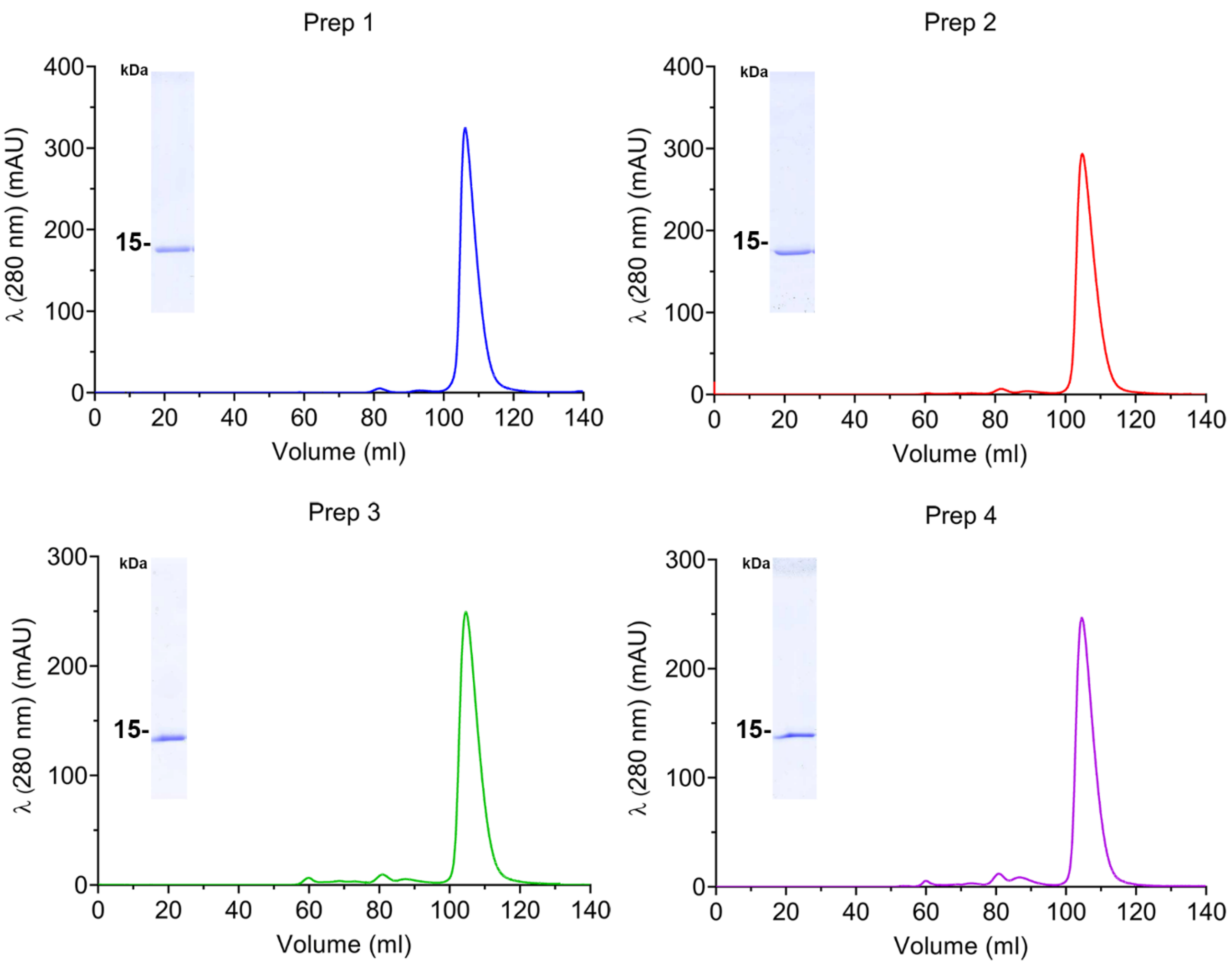

Figure S1. Isolation of CRES. Gel filtration column chromatograms and Coomassie stained SDS-PAGE gels for each of four CRES preparations.

Helix	15 ± 2
Strand	27 ± 1
Turn	18 ± 3
Unordered	41 ± 2

Figure S2. CRES secondary structure. The secondary structure of CRES was predicted from the spectral data in Fig 1d using the CONTINLL algorithm and reference data sets set 4 and SMP180 with the DichroWeb server. Data presented are the mean ± SEM from n=3 independent CRES preparations.

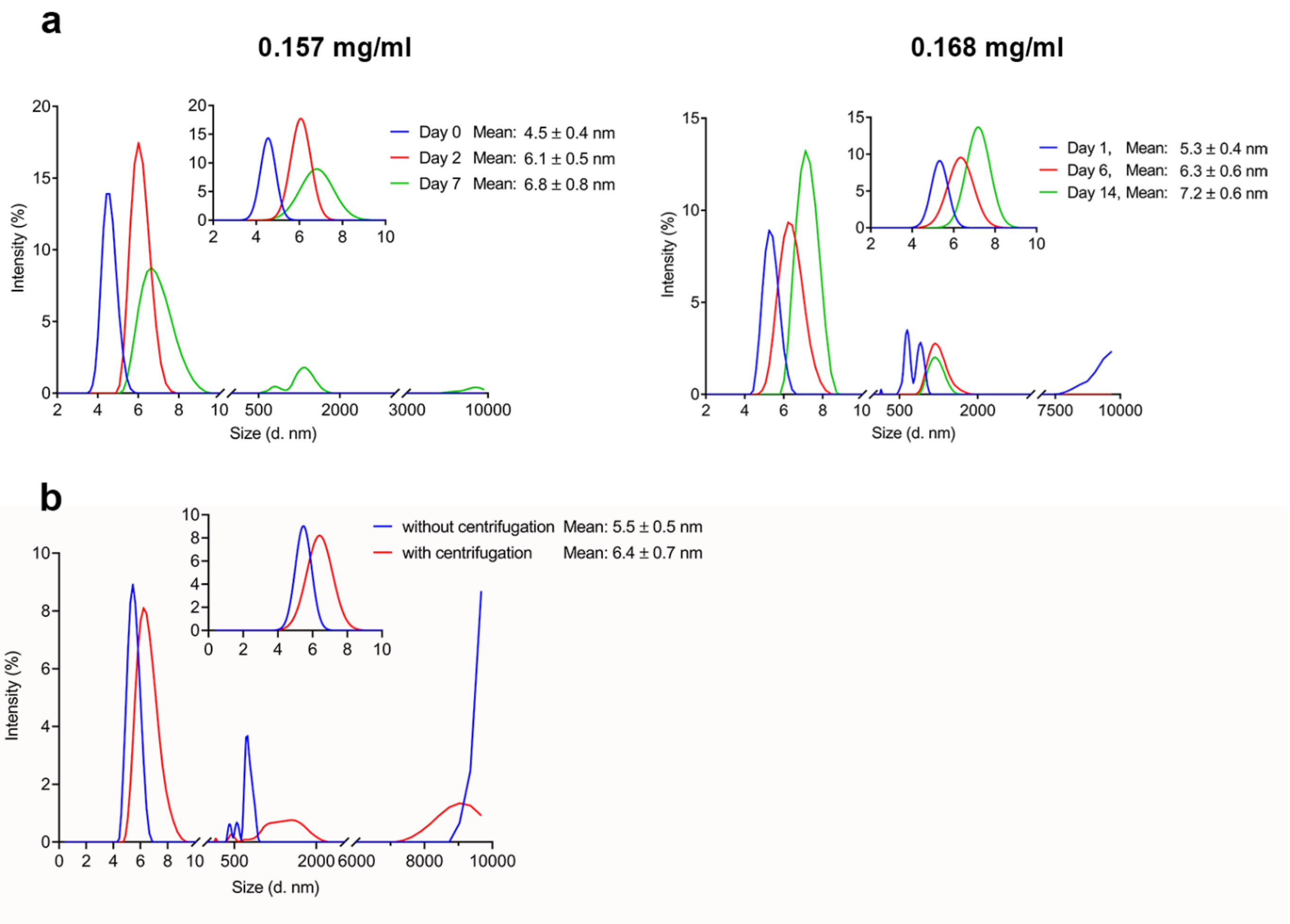

Figure S3. CRES assembles into an oligomer. a) DLS analysis was used to follow the aggregation of two freshly isolated CRES preparations (~11-16 μM) in high salt gel filtration buffer over 1-2 weeks and intensity measurements are shown. Inset, average diameter ± SD of particles in the 4-8 nm population. **b)** CRES in gel filtration buffer that underwent ultracentrifugation at 175,000 x g for 1 hour to remove preexisting aggregates assembled into an oligomer (red).

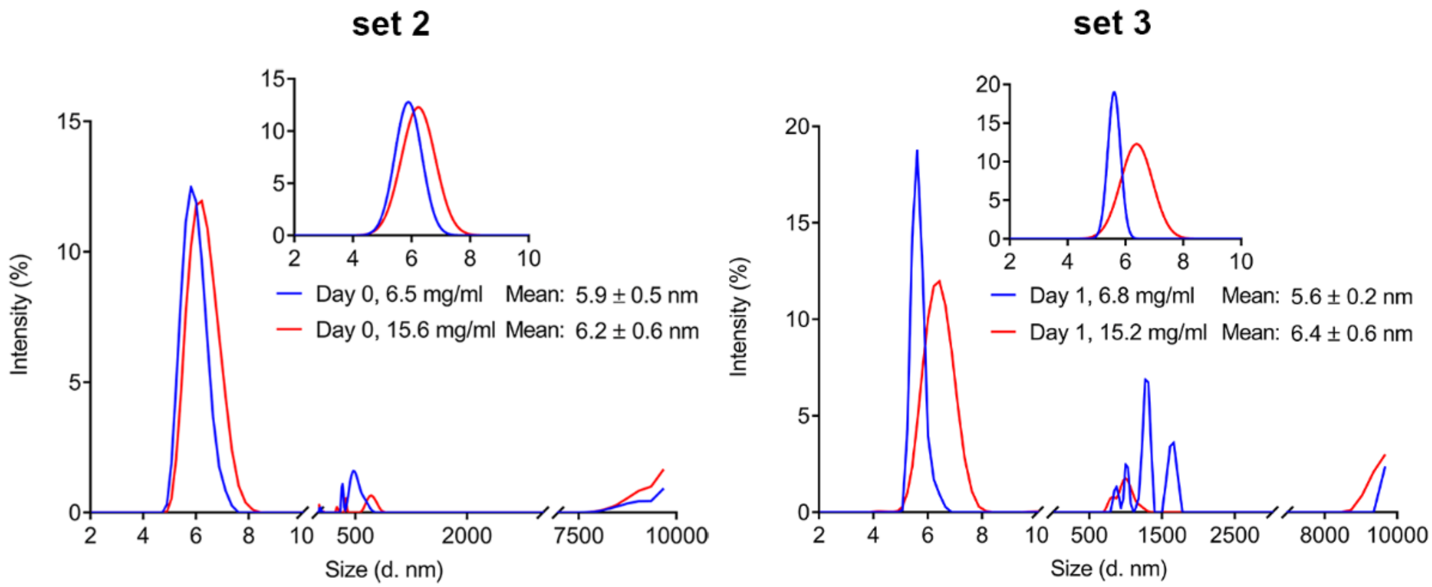

Figure S4. High concentrations facilitate the assembly of the CRES oligomer. DLS was performed within 24 hours on two CRES preparations (sets 2 and 3) following buffer exchange with 4 mM potassium phosphate buffer, pH 7.4 and concentration to mid- (6.5-6.8 mg/ml, ~0.44 mM) (blue) and high (15.2-15.6 mg/ml, ~1.0 mM) (red) concentrations. Intensity measurements showed populations of CRES particles at 4-8 nm and 500-15000 nm. Inset, average diameter ± SD of particles in the 4-8 nm population.

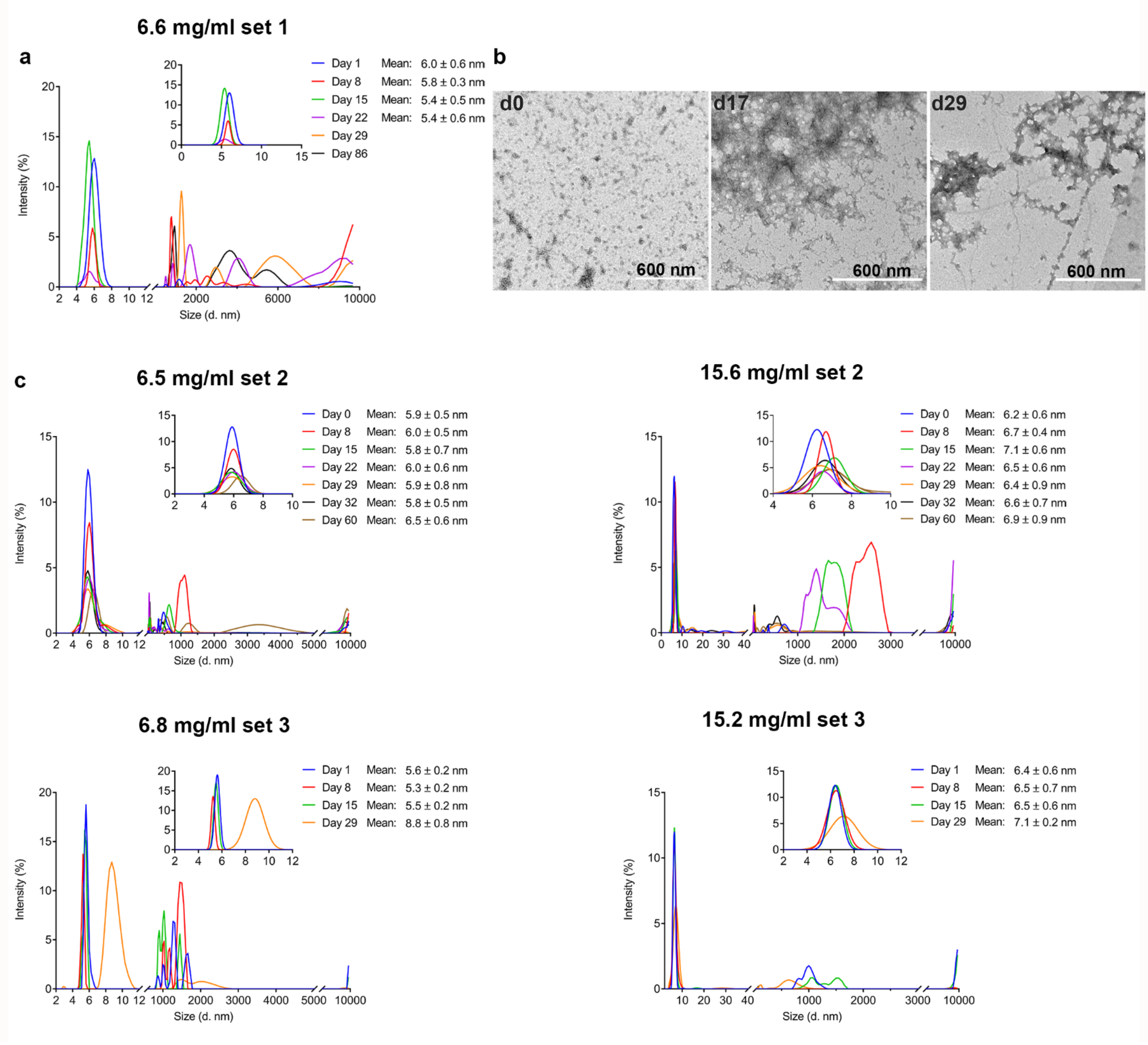

Figure S5. CRES form a stable oligomer. The aggregation of mid-concentration CRES sample (6.6 mg/ml, set 1) was followed over time by **a)** DLS analysis and **b)** Negative stain TEM. **c)** The aggregation of two additional CRES preparations (sets 2 and 3) concentrated to mid- (6.5-6.8 mg/ml) and high (15.2-15.6 mg/ml) concentrations was followed over time using DLS. **a) c)** Inset, average diameter ± SD of particles in the 4-8 nm population. No particles were present in the 4-8 nm range for days 29 and 86 in **a)**.

Helix	22 ± 2
Antiparallel	15 ± 1
Parallel	8 ± 0.3
Turn	9 ± 2
Other	46 ± 3

Figure S6. His-CRES secondary structure. CD was performed on His-CRES (0.158 mg/ml) in 2.5 mM MES, pH 5 and secondary structure was predicted from spectral data using the BeStSel server. The data are presented are the mean ± SEM from n=3 independent His-CRES preparations.

a

	W0	W1	W2	W3	W5	W6	W8	W10	W12
Helix	10	8	10	11	9	8	10	7	7
Strand	33	39	33	30	38	38	35	35	35
Turn	13	11	13	25	24	12	12	23	23
Unordered	43	42	44	34	34	42	43	34	34
NRMSD	0.14	0.22	0.12	0.09	0.08	0.08	0.10	0.14	0.15

b

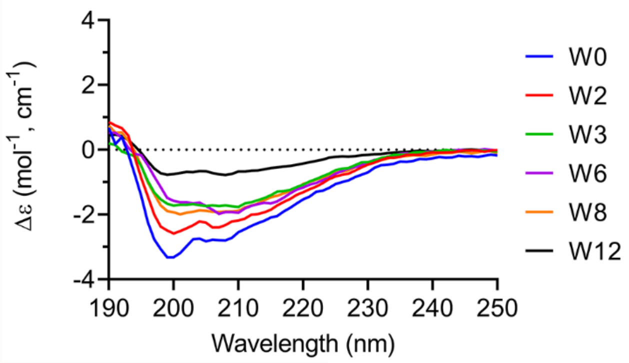

	W0	W2	W3	W5	W6	W8	W10	W12
Helix	10	11	9	10	11	9	4	0
Antiparallel	25	33	31	35	30	31	41	44
Parallel	6	2	0	2	0	1	0	0
Turn	14	13	14	12	13	13	14	14
Other	46	41	47	40	47	46	41	41
NRMSD	0.03	0.02	0.02	0.02	0.02	0.02	0.03	0.04

Figure S7. CRES structural conversion involves α-helix to antiparallel β-sheet transition. a) Secondary structure of the mid-concentration CRES sample from Fig 3 was predicted using the CONTINLL algorithm (reference data sets 4 and SMP180) with the DichroWeb server. **b)** CD spectra were collected over 12 weeks (W0-W12) from a high concentration CRES (15.0 mg/ml) sample in 4mM potassium phosphate buffer, pH 7.4. Samples were diluted to 0.157 mg/ml and spectra immediately collected. Spectral curves show experimental data. Table shows the secondary structures predicted by the BeStSel server. NRMSD, normalized root square deviation.

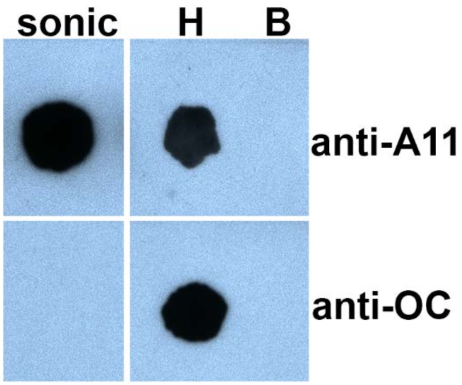

Figure S8. CRES amyloids formed by sonication bind anti-oligomeric A11 antibody but not anti-fibrillar OC antibody. Four µgs of freshly sonicated CRES (**sonic**) were spotted on to nitrocellulose and incubated with anti-oligomeric A11 and anti-fibrillar OC antibodies in dot blot analysis. **B**, buffer only. Nonsonicated His-CRES (**H**) was included as a positive control for the anti-OC antibody.

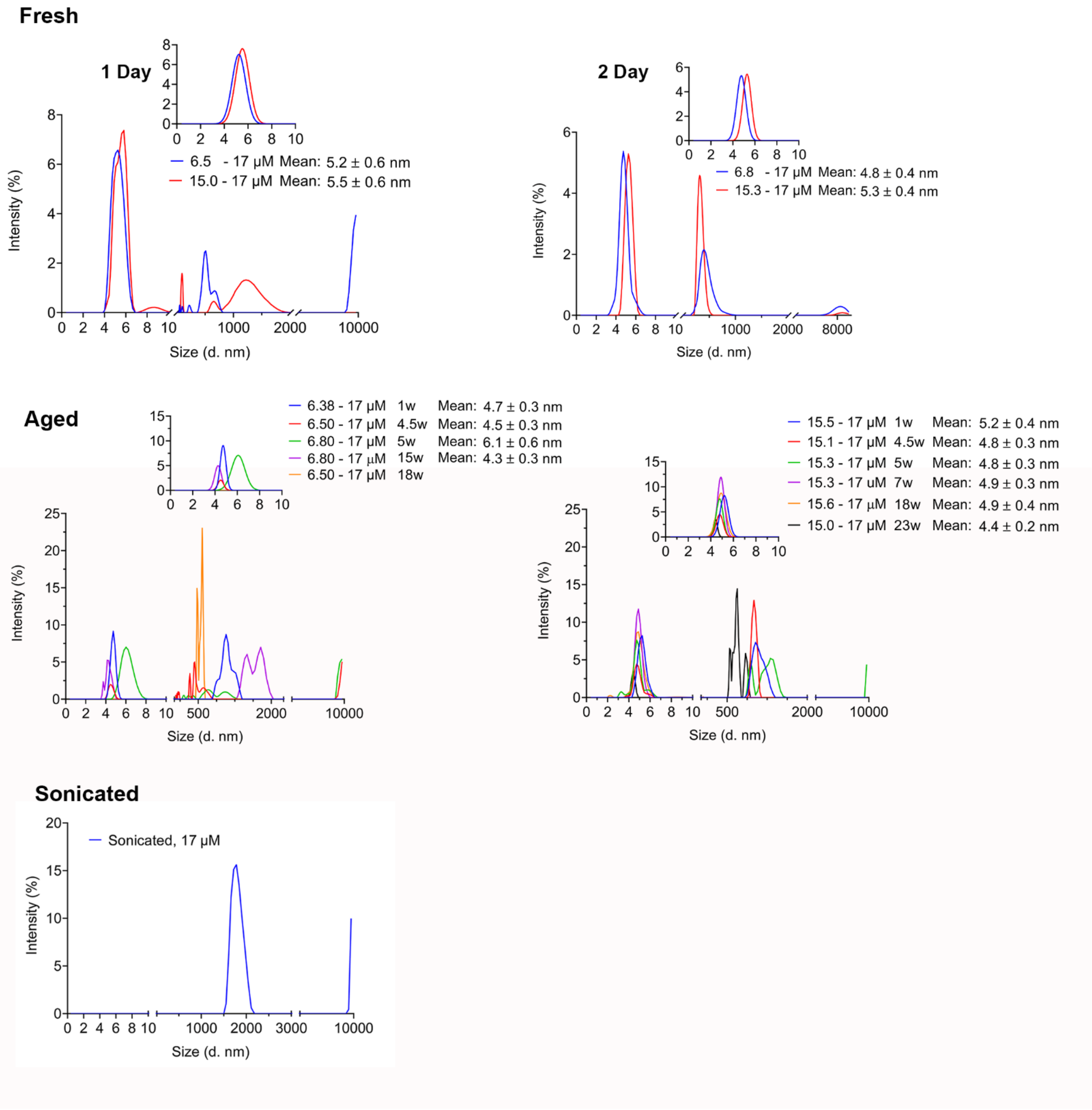

Figure S9. Dissociation of CRES following dilution. Freshly prepared (1, 2 day old) and aged (1-23 wks) mid- and high concentration CRES preparations and sonicated CRES were diluted to 17 µM and analyzed by DLS. Intensity measurements show populations of CRES particles at 4-8 nm and 10-2000 nm. Particles greater than 10000 were also present but were too large for analysis by DLS. Inset, average diameter ± SD of particles in the 4-8 nm population revealed the majority of CRES particles were monomers (4.3-5.5 nm) with an occasional oligomer (6.1 nm). In the aged mid-concentration sample after 18 weeks no particles were detected in the 4-8 nm range. Sonicated CRES only contained particles 1500 nm or larger.

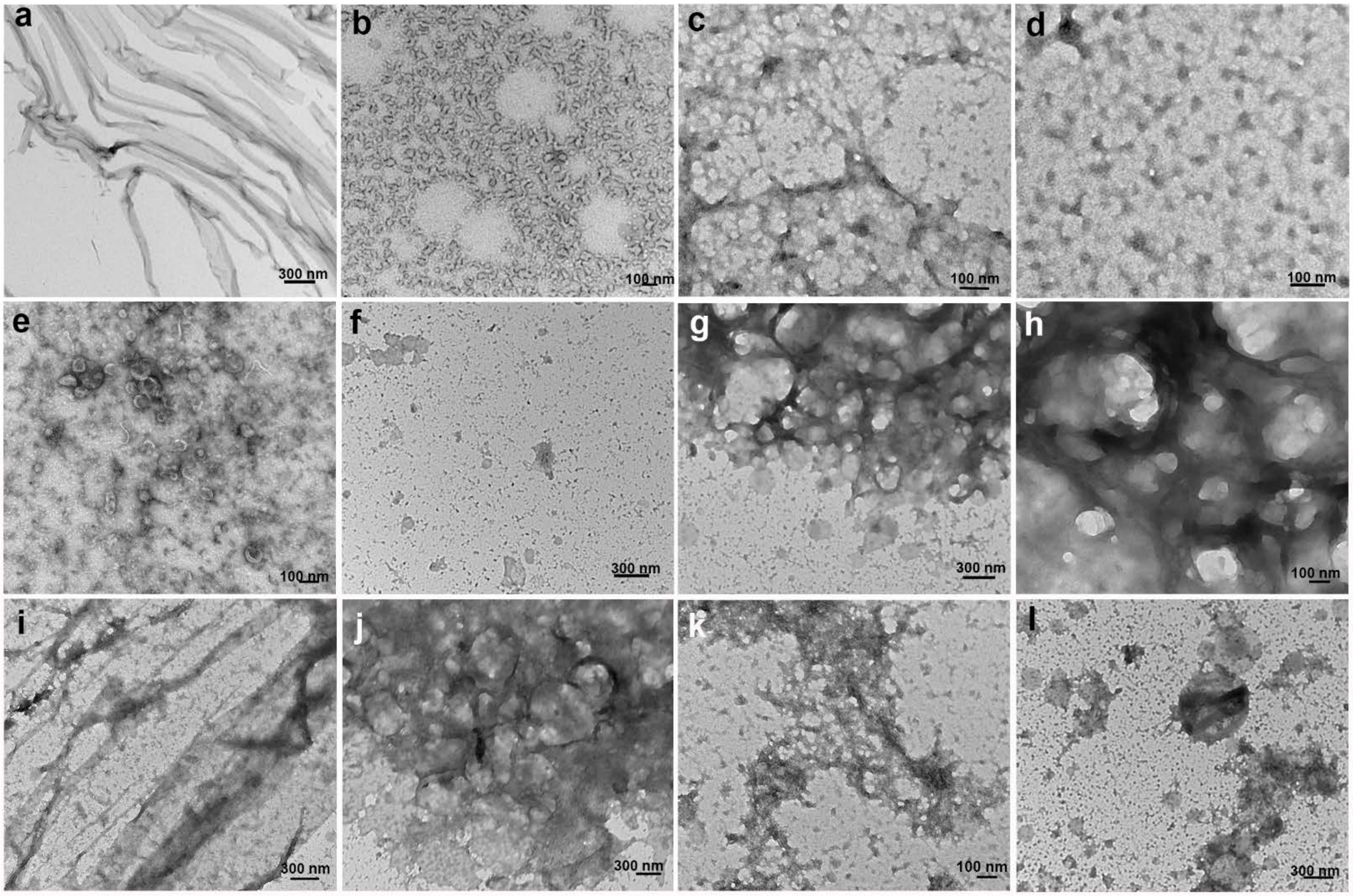

Figure S10. **Negative stain TEM of epididymal amyloid matrix/CRES seeding reactions.** Images show structures present after 24 hours in **a**, **b**, monomer; **c**, **d**, CRES WT caput epididymal seed; **e**, **f** CRES KO caput epididymal seed; **g**, **h** monomer + CRES WT caput epididymal seed; and **i** - **l**, monomer + CRES KO caput epididymal seed. Images are from the experiments presented in Fig. 8.
